# Protocol for a realist and social return on investment evaluation of the use of patient-reported outcomes in four value-based healthcare programmes

**DOI:** 10.1136/bmjopen-2023-072234

**Published:** 2023-04-27

**Authors:** Gareth Roberts, Adele Cahill, Charlotte Lawthom, Martine Price, Christopher Blyth, Carys Jones, Leah Mc Laughlin, Jane Noyes

**Affiliations:** 1 Aneurin Bevan University Health Board, Newport, Gwent, UK; 2 Public Health Wales, Cardiff, UK; 3 Bangor University, Bangor, Gwynedd, UK

**Keywords:** Health policy, Quality in health care, Epilepsy, Parkinson-s disease, Heart failure, Cataract and refractive surgery

## Abstract

**Introduction:**

There is growing recognition that in order to remain sustainable, the UK’s National Health Service must deliver the best patient outcomes within available resources. This focus on outcomes relative to cost is the basis of value-based healthcare (VBHC) and has led to interest in the recording of patient-reported outcome measures (PROMs) to measure patient perspectives on the impact of a health condition on their lives. Every health board in Wales is now required to collect PROMS as part of routine care. We will evaluate the VBHC programme implemented in a lead health board. The study aim is to understand what works about PROMs collection, for whom, in what contexts and why in a VBHC context. In addition, we will assess the social value of integrating PROMs collection into routine care.

**Methods and analysis:**

A three-stage mixed-methods study comprising a realist evaluation integrated with social return on investment (SROI) analysis across four conditions; Parkinson’s disease, epilepsy, heart failure and cataract surgery. Workstream 1: Development of logic models, informed by a scoping review, documentary analysis, patient and public involvement (PPI), staff and key stakeholder engagement. Workstream 2: Realist evaluation building on multiple data sources from stages 1 to 3 to test and refine the programme theories that arise from the logic model development. Workstream 3: SROI analysis using interview data with patients, staff and carers, stakeholder and PPI engagement, anonymised routinely collected data, and questionnaires to populate a model that will explore the social value generated by the implementation of PROMs. Findings across stages will be validated with key stakeholders.

**Ethics and dissemination:**

The study is approved by Wales Research Ethics Committee #5 (22/WA/0044). Outcomes will be shared with key stakeholders, published in peer-reviewed journals and presented at national and international conferences.

This is an open access article distributed in accordance with the Creative Commons Attribution Non Commercial (CC BY-NC 4.0) licence, which permits others to distribute, remix, adapt, build on this work non-commercially, and license their derivative works on different terms, provided the original work is properly cited, appropriate credit is given, any changes made indicated, and the use is non-commercial.

**STRENGTHS AND LIMITATIONS OF THIS STUDY**

- Realist evaluation and social return on investment methods are ideally suited to exploring what happened when patient-reported outcome measures were implemented into routine practice as part of the value-based healthcare (VBHC) programme.
- The realist evaluation and social return on investment analysis will have a high level of stakeholder involvement, including National Health Service staff, patients, carers and other organisations to provide insights not typically included via alternate study designs and methods.
- Aneurin Bevan University Health Board was one of the first adopters of VBHC in Wales and has a large repository of routinely collected data available for the analyses.
- The study is limited by time and resources to evaluating four services, nonetheless these have been chosen in order to sample maximum variation, that is, chronic and acute conditions, and conditions affecting a wide age range.

## Introduction

Countries with developed healthcare systems are struggling to meet demand and the needs of their populations.^1^ Globally, there is growing recognition that healthcare services either have or will soon become unsustainable.^2 3^ Reasons for this are complex and include, an increasingly ageing, frail and diversifying population,^4^ increasing comorbid conditions,^5^ and lack of patient involvement in interventions designed to address complexity of care and service access.^6^

Increasing spending to match demand is no longer a viable solution and accumulated evidence reinforces the view that in order to remain sustainable, the National Health Service (NHS) in the UK needs to take a different approach.^7^ One concept that is developing growing recognition, maturity and support as a potential solution is value-based healthcare (VBHC) (see glossary of terms; table 1).

**Table 1:** Glossary of terms

Acceptability	Explores the perception among stakeholders that the initiatives were agreeable, palatable and/or satisfactory. Usually assessed based on stakeholder knowledge or experience. Various dimensions of initiatives can be considered, such as the content, complexity and comfort from the perspective of different stakeholders.
Adoption	Otherwise referred to as uptake, refers to the intention, initial decision or action to implement initiatives.
Appropriateness	Refers to the perceived fit, relevance or compatibility of initiatives to the implementation setting. Considers the potential resistance to implementation efforts or alignment with care priorities from a variety of stakeholder perspectives.
Attribution	An assessment of how much of an outcome can be attributed to the programme or initiative under evaluation.
Context	Refers to the literal context in which interventions happen. Contexts can be different for different stakeholders. For example, a busy clinic would be a clinical context. A single working parent with two small children would be a patient context. Understanding the contexts in which interventions are implemented is vital to understanding (and often predicting) their effectiveness.
Deadweight	An assessment of the proportion of observed change that stakeholders would experience over the study period, regardless of taking part in value-based healthcare (VBHC) programmes.
Displacement	An assessment of the proportion of potential outcomes that are displaced by other outcomes, for example, implementing a programme to reduce crime in one area could have the unintended effect of displacing crime to a neighbouring region without the crime prevention programme.
Drop-off	An assessment of how long outcomes last into the future.
Feasibility	Explores the extent to which initiatives could be successfully applied within given settings. For example, it may have been considered acceptable and appropriate, but resourcing requirements might have made it unfeasible.
Fidelity	Considers the degree to which the initiatives were implemented as prescribed. Explores the alignment between care received by patients and the model of care developed as part of the initiative. The dimensions of focus include the adherence, quality, component differentiation, exposure to interventions and patient responsiveness/involvement.
Financial proxy	An estimate of the value of an outcome to the stakeholder experiencing that outcome.
Impact map	A spreadsheet which maps inputs, outputs and outcomes in a way which allows quantitative data to be entered to calculate the SROI ratio.
Implementation cost	Conceptualised as the direct cost impact of an implementation attempt. Three components of cost are generally considered:(1) costs of initiative care models;(2) costs of the implementation strategy used and (3) varying costs of delivery by setting.
Mechanism	Refers to the underlying entities, processes or structures which operate particular contexts, for example, a specialist nurse in a clinic using PROMs data to plan care with the patient and change a medication based on the PROMs data, or an online app designed for use by patients to access and act on their PROMS data to better self-manage their care are hypothetical examples of mechanisms. There are no limits to the number of mechanisms in a given context.
Middle range theory	A more general explanation drawn from a range of programme theories. A middle range theory could be adapted for use in many different contexts—it is not dependent on a specific disease condition.
Outcome	Refers to the results of an intervention, whether intended or not. For example, an intended outcome of routine PROM collection is reduced symptom burden for patients. Hypothetical unintended outcomes could include: a perception of surplus ‘routinely collected patient data’ exasperates clinical professionals; or highlights gaps in NHS data analysis and data linking capacity.
Penetration	Otherwise referred to as coverage/saturation, captures the integration of initiatives within settings (eg, number of initiatives adopted at a site, number of patients utilising a service, number of health professionals providing a service).
PROM	Patient-reported outcome measures. These are validated questionnaires that patients are requested to complete at various points as part of their routine care. We provide examples in the appendices.
Prudent healthcare	A Welsh Government adopted strategy defined by four overall principles: Public and professionals are equal partners through coproduction; care for those with the greatest health need first; do only what is needed and do no harm; reduce inappropriate variation through evidence-based approaches.
Programme theory	An explanation of what a particular programme of work is intended to deliver/improve/change. For example, routine PROM collection in cataracts services aims to identify patients who will benefit from surgery and use these data to triage patients to more appropriate interventions which may have better patient outcomes.
Realist evaluation	A theory-driven method used to understand the contexts, mechanisms and outcomes underpinning a programme, thereby providing an explanation of ‘what works, for whom and in what contexts’. Understanding the way that a programme operates is essential for scaling up activities.
Retroductive analysis	Retroduction refers to the identification of hidden causal forces that lie behind identified patterns or changes in those patterns. It asks the question: ‘why do things appear as they do? Retroduction uses both inductive and deductive logic, as well as insights or hunches. It involves thinking through what causal powers might be at work in producing observed patterns or changes in patterns. It is underpinned by a belief that an understanding of causation cannot be achieved using only observable evidence. Retroductive theorising requires that inquirers use their common sense, intelligence, expertise and informed imagination to build and test theories about underpinning causal processes.
Social return on investment (SROI)	A method used to measure and value outcomes that matter to the people and organisations (stakeholders) who experience them. It incorporates social, economic and environmental value, and produces an SROI ratio which tells us ‘£x of social value is generated for every £1 invested in the programme under evaluation’.
Social value	A concept which encompasses social, economic and environmental value.
Stakeholder	People or organisations directly affected by the activities of the programme under evaluation.
Sustainment	Maintenance of fidelity measurements in an ongoing, stable manner. Emphasises the integration of the initiatives within an organisation’s culture. Attaining long-term viability is considered the final stage of diffusion and dissemination within an organisation.
Theory of change	A type of logic model which describes the relationship between inputs, outputs and outcomes/contexts, mechanisms or outcomes.
Value-based healthcare (VBHC)	VBHC is an approach which shifts the focus of healthcare onto the outcomes that matter for patients. It is realised when we achieve the best possible health outcomes for our population within available resources. It is a Welsh Government Policy and is linked to all major health and social care policy contexts. One way to support VBHC are PROMs.

NHS, National Health Service.

Value in healthcare is realised when we achieve the best possible health outcomes for our population within the resources that we have available.^8^

Demonstrating high value care in ‘real-world’ settings is challenging since most healthcare systems (including the NHS) have traditionally focused on recording processes (mainly access to care) rather than patient outcomes. The emergence of VBHC as a concept has led to a renewed interest in recording patient-reported outcome measures (PROMs) as part of routine care.^9^ PROMs differ from clinician recorded outcomes since they measure patients’ perspectives on the impact of disease on their lives. To this end, PROMs have become a central feature of a VBHC approach, with organisations such as International Consortium for Health Outcomes Measurement recognising the potential benefits for patients from using these tools in routine care rather than within the confines of clinical trials.^10^

In 2015, the Aneurin Bevan University Health Board (ABUHB; an integrated Healthcare system covering a population of 600 000 in Wales, UK), set up a dedicated VBHC team with the aim of collecting PROMs data across multiple disease areas. The programme has grown exponentially and is now collecting PROMs in over 25 disease areas. In order to support collection at scale, ABUHB partnered with a software supplier (Dr-Doctor) to develop a novel electronic outcomes capture platform. The platform enables either remote or ‘in-clinic’ collection of electronic PROMs.

One of the key aims of the programme in ABUHB is to use PROMs in direct care as a tool for driving improvement in both patient care and patient outcomes. There are a number of potential mechanisms whereby collecting PROMs may achieve this as described in table 2.

**Table 2:** How routine collection of PROMs may lead to improved patient care

**Care domain**	Potential mechanisms where PROMS may improve care and outcomes
**Symptom burden**	At the individual patient level, the use of PROMs may empower patients, helping them start conversations with clinicians and focus on symptoms that matter to them. This may improve the detection of problems and support shared clinical decision-making.
**Disease trajectory**	In chronic disease settings, temporal changes in symptoms can be plotted over time enabling discussions about disease trajectory and the impact of any treatments. Such data have not traditionally been visible to patients or clinicians.
**Remote Monitoring**	The use of electronic PROMs lends itself to the development of remote monitoring, enabling virtual clinics and virtual triaging of patients awaiting treatment or review.
**Service improvement**	Feedback of aggregate PROMs data may lead to improved patient care by enabling services to identify which aspects of the service works well and which do not meet patient expectations.
**Benchmarking**	Reporting of casemix adjusted aggregate PROMs data may enable benchmarking with other organisations, thus driving improved care. Aggregate, casemix adjusted data may also enable shared decision-making by giving patients a realistic expectation of what outcomes they may achieve from a given treatment (based on aggregate data derived from similar patient cohorts).

PROMs, patient-reported outcome measures.

Despite early reported benefits, the PROMs programme within ABUHB has not been formally evaluated. It is important to undertake an evaluation since there are a number of potential barriers which may impact on the goals described above, and thus limit the utility of PROMs. These barriers may include poor completion rates by patients, competing priorities preventing busy clinicians from viewing PROMs and clinicians lacking the competence or confidence to deal with symptoms uncovered by PROMs. On a service level, resource limitations may mean that appropriate timely referrals and review by other specialties (such as mental health) may not be possible and other solutions such as social prescribing may be required. Another key challenge to PROMs collection is digital exclusion. Technology has transformed healthcare (in particular post-COVID-19); however, many people in the UK cannot access this technology. Those who are excluded are also likely to be disadvantaged socially and economically and hence likely to be the citizens who require healthcare the most.^11^

Reviewing the literature for evidence in this area is challenging since most studies of PROMs have related to their use in clinical trials or national audits rather than their use as a tool in routine care. It is clear that the growing use of PROMs in clinical practice has occurred predominantly on the belief that it is intuitively ‘a good idea’ rather than an approach that is backed by an extensive evidence base.^12^ The relatively small evidence base that exists for their use in clinical practice is based on studies undertaken almost exclusively in mental health or oncology/palliative care services. Within these services, there appears to be conflicting data as to whether PROMs support or constrain patients in sharing or raising issues with clinicians. It would appear that PROMs are useful for patients who preferred not to talk about personal or sensitive issues, thereby helping them to share information.^13^ It is also clear that some clinicians perceived that standardised PROMs constrained the patient–clinician relationship because they did not capture the complex and dynamic nature of patients’ problems.^12–18^

Given the dearth of evidence in the literature, the scale and pace at which the programme in ABUHB is progressing, as well as the ambition of Welsh Government to replicate the work across Wales, it is imperative that the programme undertaken thus far is formally evaluated. In particular, we need to fully understand (A) what is working, (B) who it is working for and (C) what are the key mechanisms to maximise the use of PROMs to realise VBHC at the levels specified in recent policy contexts.

### Aims and objectives

The aim is to undertake a realist evaluation and social return on investment (SROI) analysis of the collection of PROMS in the first adopter health board in Wales. Our objectives are to:

- Explore whether the PROMs currently collected encapsulate outcomes that matter to patients.
- Evaluate whether PROM collection improves patient care in Parkinson’s disease, epilepsy, heart failure and cataract services. Improved patient care might be as follows:
  - More timely.
  - Closer to home.
  - Direct referral to relevant health professionals.
  - Avoid unnecessary hospital visits.
  - Prevent unplanned admission.
- Identify potential small-scale changes as part of continuous improvement, including service redesign, and improved use of healthcare utilisation.
- Measure the social value of integrating PROMs in routine data collection.
- Develop logic models identifying the inputs required for clinicians to use PROMs in decision-making, the context, mechanisms of change and the potential intended/unintended impacts.
- Better understand and develop ways to overcome any barriers associated with electronic PROMs collection, in order to avoid excluding cohorts of people, that is, explore whether the shift to digital collection of PROMs excludes some communities, thus widening healthcare inequalities.

### Research questions

- What works about PROMs collection, for whom, in what contexts and why in a VBHC context?
- What is the SROI of integrating PROM collection in routine care in a VBHC context?

## Methods

**Figure 1:**
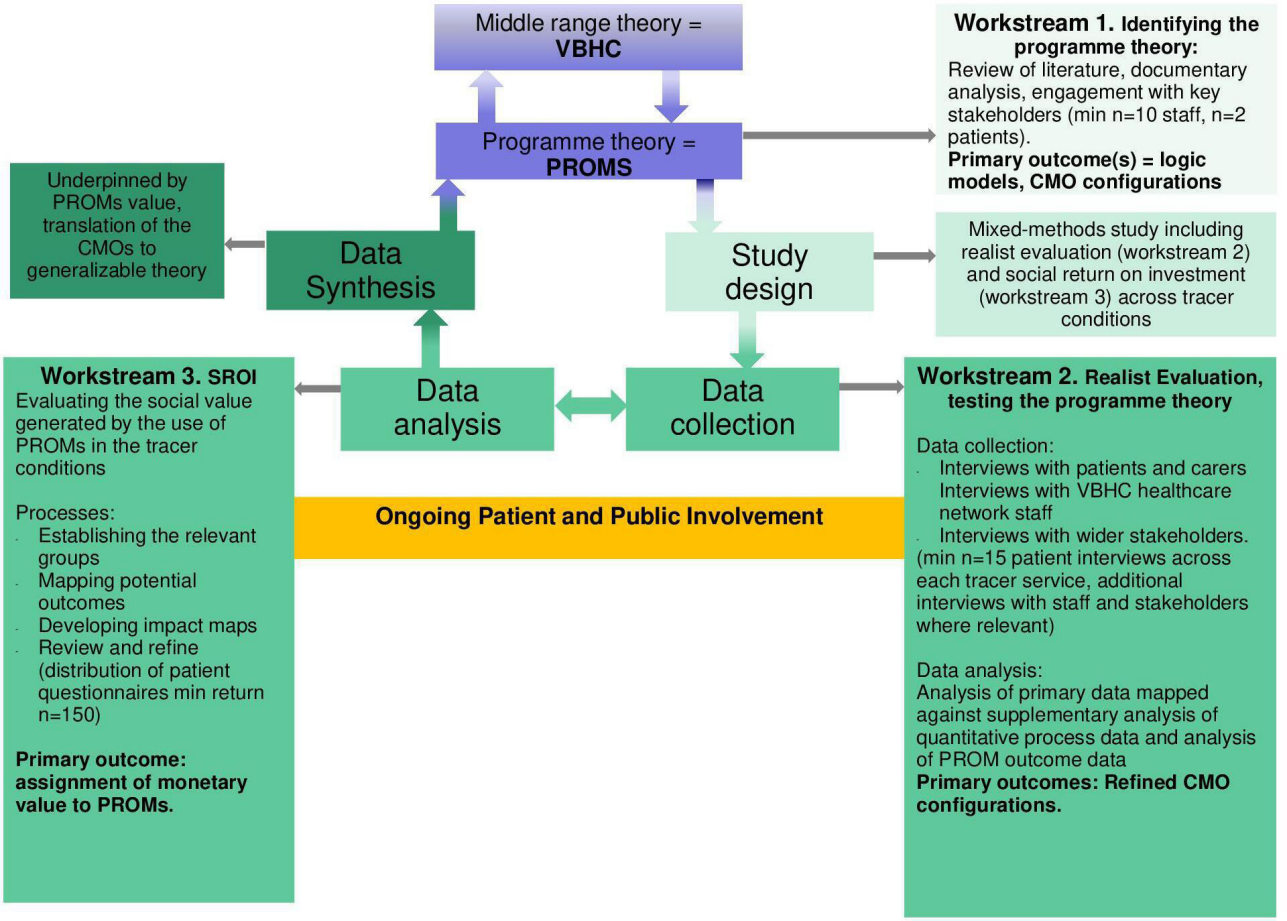
Outline of realist evaluation with SROI integration and explanation of processes. SROI, social return on investment.

A mixed-methods study comprising a realist evaluation and SROI analysis. The realist and SROI analyses are complementary and will be undertaken in tandem with several key points for integration built into the study design. Combining the approaches will help learn more about what is needed to achieve the goals of VBHC at scale. The study design, overarching processes and integration are illustrated in figure 1.

### Setting

Health services for people with cataracts, epilepsy, heart failure and Parkinson’s disease will be included (online supplemental file 1). These are four service areas where PROMs have been routinely collected for a number of years. By choosing four diverse tracer services, we will be able to include:

10.1136/bmjopen-2023-072234.supp1 Supplementary data

- A surgical intervention (cataracts).
- A chronic disease with a large cohort of young adults (epilepsy).
- A chronic disease affecting a predominantly elderly and sometimes frail cohort (Parkinson’s disease).
- A long-term chronic condition that is most common in older people, but can affect people at any age (heart failure).

The sample across the four tracer services is suitably big enough to address the overall research aims and manageable enough to achieve the objectives within the budget and time constraints. The individual services and their adaption of PROMS to date are described in more detail in online supplemental file 2.

10.1136/bmjopen-2023-072234.supp2 Supplementary data

#### Data collection and analysis

Work stream 1 (WS1): Scoping review, documentary analysis and stakeholder engagement to develop logic models and refine programme theories for the realist evaluation and SROI analysis.

Scoping review: Primary searches will be conducted in CINAHL, Cochrane CENTRAL, EMBASE, MEDLINE, PsycINFO, PubMed and Web of Science, and we will include relevant reports found via key word searches on Google Scholar. We will liaise with partners in ABUHB to identify additional relevant literature. To minimise the risk of bias, the quality of papers will be assessed using a standardised checklist such as the AACODS checklist for grey literature^19^ or the CASP checklists for primary studies.^20^ Framework synthesis methods will be used to organise findings.

Documentary analysis: We will undertake content analysis^21^ using NVivo of the VBHC service specification, change and implementation documents (eg, PROMS data, administrative patient data, audits, evaluation surveys, clinical processes and outcomes) for Parkinson’s disease, epilepsy, heart failure and cataract surgery services. We will identify the key elements needed to develop initial programme theories and logic, and build an understanding of implementation processes for each tracer service.

Engagement with key stakeholders such as VBHC steering group members, programme designers, clinical implementers and patient and public involvement (PPI) representatives will also inform the development of the initial logic models and programme theories.

Through undertaking WS1, we will uncover the underlying system dynamics (relationships between agents, their attributes and rules of behaviour, network structure, feedback loops) that influence implementation and bring about outcomes. The initial logic models and programme theories will be further developed as the study progresses and serve as the theoretical framework for the study.

Workstream 2 (WS2): Realist evaluation

We will follow the methods of Pawson^22^ and Rycroft-Malone *et al*^23^ and focus will shift from developing initial theories in WS1 to testing theories and refining the narrative that best explains the implementation of VBHC and observed outcomes for people in the tracer services. We will explain the circumstances (context) and mechanisms that drive outcomes (CMOs) in each tracer service. CMOs will be mapped against the predetermined programme theory to determine what is and is not working and the mechanism of action to achieving the observed outcomes (positive and negative).

We will continue to engage with key stakeholder groups and PPIs.

We will conduct realist exploratory semistructured interviews with key people in implementing PROMs such as VBHC network staff, local healthcare professionals in the tracer services, patients and carers, and combine with quantitative process data. PROMS outcome data and how they are valued will be used to test and refine CMOs constructed. We will also ask patients for their NHS number to cross-check against anonymised PROMS data to identify if they have received and returned PROMS as anticipated. Interviews will be recorded, transcribed and uploaded into NVivo for analysis.

Consistent with realist approaches, analysis will be retroductive in that it will oscillate between an inductive and deductive logic to multiple data sources as well as incorporating the researchers’ own insights and PPI/stakeholder views of what causes something to work (causation) for the programme theories. We will read through and systematically analyse each interview transcript or fieldnote and code data of interest that helps explain why and how something is working or not in a specific context. Each stage of the iterative realist analysis will become progressively focused on CMO configurations. Overall, data will be analysed within data sources (documents, interviews, process and outcomes, and service evaluation data), and with key stakeholders, PPIs and steering group members over time, and then explanations will be developed across the data, with attention to the realist task of uncovering contingencies and conditions, that is, the relationships between factors that explain ABUHB’s approach to implementation of PROMs, and the conditions in which they operate. We will then revisit the programme theories and refine these in light of CMO data to confirm or disconfirm our CMOs, ensuring a diversity of data sources and another level of clarification. We will map the actual process of implementation (work as done) compared with how it was planned (work as imagined). We will also translate CMOs into generalisable theoretical models (middle-range theories) for implementing large-system, VBHC programmes. The process will begin by presenting and defining CMOs with key stakeholders. Further testing will be undertaken by explicitly seeking disconfirming or contradictory data and considering other interpretations that might account for the same findings.

We will review the realist findings for the tracer services (including programme theories and CMO configurations) ‘vertically’ to identify common thematic elements according to CMOs. Data will also be analysed across each service ‘horizontally’ to uncover potential generative causal patterns between mechanisms and outcomes. This process will potentially translate the specifics of implementing VBHC in the tracer services to more analytically driven generalisable theories for scaling up the benefits from these care delivery models to achieve large system transformation across health boards in Wales and beyond.

Workstream 3 (WS3): SROI analysis

We will develop an overall programme-level theory of change to establish how inputs (eg, costs, staffing) are converted into outputs (eg, numbers of patients seen), and subsequently into outcomes that matter to stakeholders affected by the programme (eg, improved mental health). The social value generated by these outcomes is then estimated in a similar way to cost–benefit analysis.

SROI analysis will be operationalised through the stages outlined in the guide to SROI analysis^24^:

1. **Establishing scope and identifying stakeholders (carried out in WS1**)
  - The scope of the study is to evaluate the social value generated by the use of PROMS in the VBHC programmes for the four services. Stakeholder involvement is critical to both the design and conduct of the study; particularly in relation to the development of programme theories. To identify stakeholders, we will list all potential groups who might affect or be affected by the activities of the programmes listed above.
2. **Mapping outcomes**
  - The next step is to identify the potential outcomes of each programme (positive or negative, intentional or unintentional). Informed by the documentary analysis and logic models developed in WS1, an impact map will be created for each service to explain the relationship between programme inputs, outputs and outcomes for each stakeholder group, and how these outcomes can generate value.
3. **Evidencing outcomes and giving them a value**
  - Longitudinal data on PROMs and clinical outcomes will be extracted at an anonymised, aggregated level allowing us to evaluate the relationship between PROM collection and the outcomes experienced by patients. Data will be aggregated by age, gender and clinical severity. A benefit of using routinely collected data is that it facilitates access to a larger cohort than would be possible with prospective data collection, thus reducing research time and costs.
  - It will be important to define what will be considered a material change for each of the outcomes being measured. For example, increased physical activity may be an outcome that patients experience as a result of their treatment; however, a material change in physical activity could be defined as a patient crossing the threshold from not meeting, to now meeting, the NHS recommendation of undertaking 150+ min of moderate intensity activity per week. Once the magnitude of change experienced by each stakeholder group has been identified through a combination of routinely collected data; the next step of the SROI analysis involves assigning a monetary value to the outcomes experienced by stakeholders using a financial proxy.
  - Outcomes occurring beyond 1 year will be discounted by 3.5% per annum to minimise the risk of overclaiming the amount of social value generated by the programmes.
  - The cost of inputs required to deliver PROMs in the tracer services will be identified in consultation with VBHC leads at ABUHB.
4. **Establishing impact**
  - Establishing impact is necessary to reduce bias and the risk of overclaiming the benefits of the programme. We will include model parameters to take into account deadweight (the proportion of observed outcomes that would have happened to stakeholders without PROMS); displacement (the proportion of outcomes that have been displaced from one sector to another); attribution (the amount of observed outcomes that can be direct attributed to the programme) and drop-off (the length of time that outcomes last for stakeholders). The model inputs for these variables may vary between stakeholder groups, so values will be established through stakeholder interviews and routinely collected data.
5. **Calculating the SROI**
  - Microsoft Excel will be used to create a model for running the SROI analysis. The model variables will be identified through the logic models developed in WS1 and further evolved in WS2. The parameters for attribution, deadweight, displacement and drop-off will be derived from analysis of routinely collected data and varied in a range of one-way sensitivity analyses. The model will be populated with data obtained through the extraction of routinely collected data. The SROI ratio is calculated by dividing the total value of outcomes by the total value of inputs across all stakeholders. The resulting ratio is the amount of social value generated for every £1 invested in the programmes. In addition to calculating the base case scenario, we will perform a range of sensitivity analyses to explore how the SROI ratio would be affected if various input parameters were changed, different financial proxies were used and varying levels of outcomes were achieved to those observed in the base case. A checklist for quality assessment in SROI analysis^25^ will be used as a framework to guide the reporting of the findings.

### Identification of routinely collected data for sharing in WS2 and 3

We will identify what routinely collected data of specific interest related to VBHC and PROM implementation in the four services (eg, PROMS data, administrative patient data, audits, evaluation surveys, clinical processes and outcomes) could be used in the realist evaluation and SROI. We will then develop a process to securely share these anonymised data between ABUHB and the research team.

### Sampling

There is no minimum sample size for realist evaluation or SROI analysis as these types of studies are primarily used to develop explanatory theory rather than to detect statistical significance. Therefore, we will interview a minimum representative sample of 15 patients and carers (separately or together) from each of the four tracer services in epilepsy, Parkinson’s disease, cataract surgery and heart failure (minimum 45 interviews), and up to 10 staff members responsible for implementing PROMs in routine practice from each of the tracer services.

### Recruitment strategy

#### Patients, carers and consultees

We will purposively select patients according to their age, gender, ethnicity, service and whether they completed a PROM or not. We may add additional attributes and classifications to the sampling frame as appropriate. Patients will be contacted by ABUHB staff in the first instance and carers will be recruited through the patients. Routes to recruitment include:

- ABUHB will send out an invitation and study information via their internal PROMs platform.
- Routine appointments or via email, or post, including patients who are unable to access PROMs online (at home) and have the option of filling in PROMs within the clinic (supported by staff).

Staff members can support potential participants to complete and return a consent to contact form or the potential participants can self-complete and return in prepaid envelopes. For participants lacking mental capacity, a consultee will be appointed. Once a consent to contact form is received, the research team will follow up to organise an interview.

#### Staff

Staff involved in the VBHC programme generally or in the four services specifically will initially be approached by ABUHB staff via meetings, direct mails and face to face contact. Purposive sampling and snowballing will be used to identify respondents best placed to provide information on PROM implementation and outcomes.

Inclusion criteria

- Stakeholders involved with the implementation of PROMs and VBHC programmes in ABUHB and where relevant other health boards in Wales.
- All professionals in ABUHB involved in the routine collection of PROMs in the tracer services.
- Patients aged over 18 in the identified services.
- Carers over 18 linked to a patient who is receiving care in the identified tracer services.
- Identified consultees for people who lack capacity to consent.

Exclusion criteria

- People under 18.
- People who do not have the capacity to consent to take part in the research and for whom a consultee is not available.

### Patient and public involvement

The study was developed alongside a wide range of PPI stakeholders involved in ABUHB, the VBHC programme, third sector and specific individuals and groups representing the tracer services. Going forward, partner organisations will have a key role in the interpretation and application of findings ensuring that outcomes have relevance and are accessible to specific needs and circumstances of under-represented or especially vulnerable groups. This will be important as we know that PROMs have the capacity to help those with the greatest need first, but these individuals may encounter the most barriers to complete a PROM, for example, disability, health literacy or socioeconomic status. Our partners can help advise on the ways identified CMOs chains may need to be modified or adapted to account for the needs of these patients in Wales.

**Figure 2:**
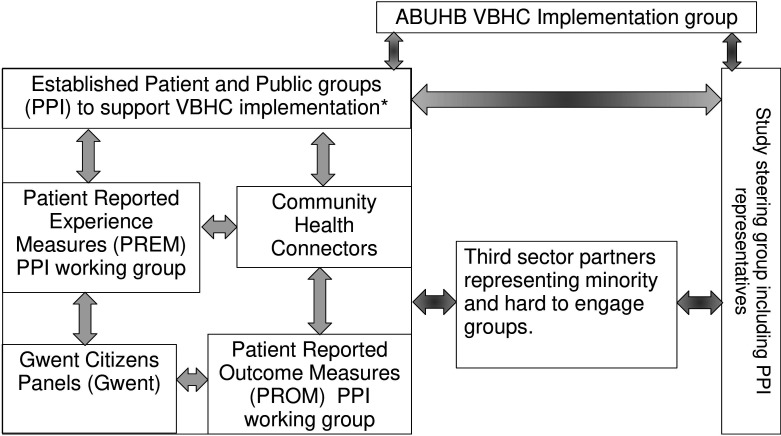
Map of PPI partners and their proposed ongoing involvement throughout the study and their potntial capacity to influence scale-up. PPI, patient and public involvement. *Every health board in Wales will have PPI structures and working groups.

Figure 2 maps and helps to visualise opportunities for PPI involvement and engagement with the multiple groups already established as part of the overall VBHC programme and how we imagine PPI to help support adoption and scale up of the study outcomes. We will follow the UK standards for PPI throughout.^26^

### Ethical issues

The study has full approval from Health and Care Research Wales Research Ethics Committee #5 (22.WA/0044). Box 1 outlines the ethical issues we have considered in designing this study.

Box 1 **Key ethical issues relevant to this study**

**Capacity to consent and personal consultees**

Informed consent will be sought from all participants. Where there are concerns over capacity to consent, thorough discussions will be undertaken within the research team and a final decision will be made by the lead researcher regarding including participants who lack capacity (either temporarily or permanently). Where participants are found to be lacking capacity, a personal consultee may be appointed.

Large print versions of documents such as study information sheets and consent forms will be available for participants with a visual impairment (eg, those attending the cataracts service). We will also provide audio recordings and explanations of the study and consent processes.

**Risk of distress to participants and pathways to referral if concerns are raised**

The research team acknowledge that taking part in this study will mean that participants will have to think about and reflect on their experiences, which may raise some concerns for individuals. Should participants be concerned by the subject matter or by any issues raised, staff participants will be signposted to consult with their line manager or occupational health department for support and patient participants will be signposted to the relevant health professional. The research team have detailed distress protocols to follow in these situations and are experienced in collecting sensitive data around personal experiences of healthcare support and living with long-term health conditions.

**Potential identification of poor clinical practice and unmet need**

Researchers are experienced healthcare scientists. Standard data protection and confidentiality protocols will be followed. We will only deviate from these if we recognise a clear and immediate risk to the health and well-being of participants. We will partner with wider social support services and signpost to these as part of the disengagement process we may also ask participants if they would like us to send a letter to the relevant healthcare professional alerting them to the fact that they have taken part in the study.

**Maintaining confidentiality of professional participants**

Professionals will be provided with an information sheet and consent form explaining that their participation is anonymous. If there is a risk of identification, for example, small team, recognisable quotes, we will group participants under ‘professionals’ and work to remove any content that may lead back to any one individual. Healthcare professionals working on the study are aware that there is no intention to identify participants at an individual level and rather to present information in general terms to facilitate learning and professional practice.

Dissemination and impact

As a coproductive study involving patients, clinicians, third sector partners and academics, findings will be shared on a continual basis. Outputs will be disseminated widely through patient and clinical networks, policy and academic routes. Consistent with realist and SROI methodology, we will disseminate findings to stakeholders as they become available through presentations, meetings and events.

We will produce a lay report to include infographics and visuals supporting wider understanding of the VBHC strategic ambition.

We speculate that the outputs of the study will include:

- A deeper understanding of the impacts of the switch to online PROMs specific to the Welsh population.
- A series of programme implementation theories explaining what is needed to realise VBHC at scale.
- A final report which will include examples of the contextual barriers and facilitators that promote the uptake of PROMS, thus enhancing understanding of how to implement them successfully in other health boards.
- An understanding of how social value is generated to different stakeholders, leading to a greater understanding of how best to optimise services to reduce inefficiencies, improve outcomes and maximise value.
- An upskilled, adaptive and responsive workforce with greater understanding of the impact and value of incorporating PROMs data in their decision-making; a workforce equipped to handle the ever-evolving role of healthcare professionals.

## Supplementary Material

Reviewer comments

Author's manuscript
