# Supplementary material for: Protocol for a realist and social return on investment evaluation of the use of patient-reported outcomes in four value-based healthcare programmes
Source: BMJ Open. 2023 Apr 27;13(4):e072234. doi: 10.1136/bmjopen-2023-072234 (PMC10151973; doi:10.1136/bmjopen-2023-072234)
Supplement: Supplementary data [file bmjopen-2023-072234supp001.pdf]

Supplementary File 1. List of PROMS used for each tracer condition

List of PROMS used for each tracer condition

Heart failure
The Patient Health Questionnaire-2 (PHQ-2). https://pubmed.ncbi.nlm.nih.gov/14583691/

Cardiomyopathy Questionnaire (KCCQ-12).
KCCQ 12 https://www.fda.gov/media/108301/download

Epilepsy
Patients Global Impression of Change (PGIC), https://pubmed.ncbi.nlm.nih.gov/14739871/

Hospital Anxiety and Depression Scale (HADS), https://pubmed.ncbi.nlm.nih.gov/6880820/

Cataract
Cataract Questionnaire - Catquest-9SF,
https://eandv.biomedcentral.com/articles/10.1186/s40662-020-00220-4

Parkinson's Disease
Part I: Non-Motor Aspects of Experiences of Daily Living (nM-EDL),
https://pubmed.ncbi.nlm.nih.gov/23607783/

Part II: Motor Aspects of Experiences of Daily Living,
https://pubmed.ncbi.nlm.nih.gov/23791519/

The Parkinson's Disease Questionnaire (PDQ-39),
https://pubmed.ncbi.nlm.nih.gov/9351479/
