# Supplementary material for: Protocol for a realist and social return on investment evaluation of the use of patient-reported outcomes in four value-based healthcare programmes
Source: BMJ Open. 2023 Apr 27;13(4):e072234. doi: 10.1136/bmjopen-2023-072234 (PMC10151973; doi:10.1136/bmjopen-2023-072234)
Supplement: Supplementary data [file bmjopen-2023-072234supp002.pdf]

Appendix. 2 Further explanation of the four tracer conditions and their implementation of PROMS to date

Description of the overall VBHC Programme in the adopter Health Board, and the tracer services

Initiative	Patient population	Description
Adopter health board (Aneurin Bevan UHB) implements VBHC from 2015 to present. 26 disease conditions included to date.	Health board serves approx. 639,000 patients; approx. 21% of total Welsh Population	Value Based Health Care: puts a focus on the value of outcomes achieved for patients. Value in health care is realised when we achieve the best possible health outcomes for our population within the resources that we have available.
**Tracer services**		
Epilepsy routine collection of PROMs 2017 to present	588 PROMs completed with an average completion rate of 51%	Identifying, quantifying and managing the burden of mood disorder. Identify relationship between patients mood, seizure frequency and medication to inform future treatment options
Parkinson's disease routine collection of PROMs 2017 to present.	840 PROMs completed with an average completion rate of 42%	Using PROMs to help deliver a more efficient and effective service based on disease severity and patient needs.
Heart Failure routine collection of PROMs 2018 to present	927 PROMs completed with an average completion rate of 85%	Using PROMs to reorganise the service more efficiently to reduce nurses' caseloads, reduce waiting times for patients and to focus treatment on patients' needs
Cataract routine collection of PROMs 2017 to present	3600 PROMs completed with an average completion rate of 88%	Using PROMs to redesign the referral and triage process, freeing up capacity and improving outcome by treating according to need rather than waiting times.

More detailed explanation of PROM integration to date

Tracer service 1: Cataracts

Cataract procedures are the most common operation performed in the UK, with more than 300,000 procedures carried out each year. The aim of cataract surgery is to improve quality of vision for patients and allow patients the ability to undertake everyday tasks. ABUHB receives 6000 cataracts referrals and performs more than 3,000 cataract operations every year. Demand for cataract surgery has exceeded capacity. Within ABUHB, a pilot study identified potential variation in pathways and outcomes. The VBHC team worked with the ophthalmology directorate to set up systematic collection of both clinical outcomes and PROMs. Visual acuity (ability to read letters of decreasing size) from a set distance was used as the clinical outcome. A cataract questionnaire consisting of 9 questions (CatQuest-9SF tool) was used as the validated PROM. The Catquest questionnaire explores visual impairment from a patient's perspective by asking questions about the ability to recognise faces, read newspapers or undertake hobbies. Preliminary data analysis of the first 600 cases suggested that (based on PROM scores) up to 20% of patients did not actually seem to benefit

Appendix. 2 Further explanation of the four tracer conditions and their implementation of PROMS to date

from surgery i.e. their visual disability did not subjectively improve after cataract surgery. Within this cohort, pre-operative PROMs scores seemed a good predictor of who would benefit the most from surgery. This has led the service to consider how pre-operative PROMs could support triage to appropriate services and inform shared decisions about surgery. We also need to better understand from the perspective of the patient and their carers what outcomes are of importance to them and whether the appropriate outcomes are being measured.

Tracer service 2: Epilepsy

The adult Epilepsy Service in ABUHB serves a population of over 6000 people with epilepsy and provides an open access service so that patients with this unpredictable condition can receive timely and responsive care. Patients can access this service via telephone or email and most patients are responded to within one working day. The service has long been aware of the rates of unmet mental health needs in the epilepsy population. There is clear data linking all forms of poor mental health with increase in seizures [23], [24]. The implementation of VBHC in ABUHB was undertaken with the aim of using PROMs to better identify and quantify the extent of anxiety and depression in people with epilepsy. Additionally, the team wanted to improve the management of mental health at individual patient level and also to track changes over time to assess whether epilepsy medication was positively or negatively affecting mood.

The Epilepsy PROM comprises questions on seizure frequency and epilepsy drugs, plus mood scores and a patient global impression change. Initial feedback from people with epilepsy revealed that the patients were overall, very happy to be asked about mood but expected feedback to them about that data and about what should happen next. The high rate of markers for suicidality uncovered by utilising PROMS scores in people with epilepsy was a deeply troubling (but potentially lifesaving) finding, but it is unclear at this stage if patient outcomes have improved.

The COVID pandemic forced the service to move to remote consultation in up to 95% of cases. COVID was also associated with an increasing demand for epilepsy services with the vast majority of people (with epilepsy) suffering with poor mood.

In terms of acting on PROMs data, in the past year the service has made:

- 18 referrals to the Mental Health team,
- 34 referrals to the Online Cognitive Behavioural Therapy (CBT) system (Silver cloud)
- 112 have been referred to online and application based mindfulness tools.
- A further 28 people have been commenced on antidepressant therapy.

PROMs are subsequently used to assess the benefits of these interventions. Please see Appendix 4 for case studies. At a local level the potential benefit to patients is clear in addition to potentially realizing the aims of VBHC, but there has been no formal evaluation to determine the mechanisms or patient outcomes and this needs addressing.

Tracer service 3: Parkinson's Disease (PD)

Parkinson's disease is a progressive nervous system disorder that affects movement. Tremors are common, but the disorder also commonly causes stiffness or slowing of movement. Within ABUHB, PROMs have been routinely collected in the PD clinic for a number of years, indeed PD was the first service to start collecting PROMs in ABUHB. The PROMs were collected in person at attendance in clinic with the PD specialist nurse, who had sight of the scores before seeing them. As well as the individual benefit of capturing all of the patient concerns, the PROMs data enables categorisation of people with PD into newly diagnosed, moderately affected and complex PD. It became clear that these patient groups had different health and social care needs with the more complex patients requiring a number of additional services

Appendix. 2 Further explanation of the four tracer conditions and their implementation of PROMS to date

such as physiotherapy and speech and language therapy. This led the service to begin overhauling the traditional clinic structure so that more complex patients could be stratified into a dedicated multidisciplinary clinics where their needs could all be addressed. Unfortunately, this development was paused during the COVID pandemic therapy which, as in other areas led to a wholescale switch to remote (rather than in-clinic) collection of PROMs. Of note, in spite of concerns that a more elderly cohort may not embrace remote collection, completion rates have been surprisingly high at 54%. A largescale evaluation is now needed to explore these issues and service overhaul in depth to see if patient outcomes have improved.

Tracer serviced 4: Heart Failure

Heart failure is a long-term condition that tends to get gradually worse over time. It cannot usually be cured, but the symptoms can often be controlled for many years. PROMs were introduced in the Heart Failure service in 2018, because the service was overwhelmed with patients and unable to meet demand. Patients were seen in an untimely manner and not necessarily in relation to their symptoms. The service introduced PROMs in an attempt to be more patient-focused. Previously, PROMs were applied at one hospital site using non-clinical staff to administer, but for a variety of reasons this was not sustainable. However, since the introduction of PROMS in the service, the service has developed a broader picture of care at a patient-level, of service improvement and of understanding the population needs of the service.
