# Supplementary material for: Protocol for a realist and social return on investment evaluation of the use of patient-reported outcomes in four value-based healthcare programmes
Source: BMJ Open. 2023 Apr 27;13(4):e072234. doi: 10.1136/bmjopen-2023-072234 (PMC10151973; doi:10.1136/bmjopen-2023-072234)
Supplement: Author's manuscript [file bmjopen-2023-072234.draft_revisions.pdf]

BMJ Open is committed to open peer review. As part of this commitment we make the peer review history of every article we publish publicly available.

When an article is published we post the peer reviewers' comments and the authors' responses online. We also post the versions of the paper that were used during peer review. These are the versions that the peer review comments apply to.

The versions of the paper that follow are the versions that were submitted during the peer review process. They are not the versions of record or the final published versions. They should not be cited or distributed as the published version of this manuscript.

BMJ Open is an open access journal and the full, final, typeset and author-corrected version of record of the manuscript is available on our site with no access controls, subscription charges or pay-per-view fees (http://bmjopen.bmj.com).

If you have any questions on BMJ Open's open peer review process please email info.bmjopen@bmj.com

BMJ Open

A Protocol for a Realist and Social Return on Investment Evaluation of the use of Patient Reported Outcomes in Four Value Based Healthcare Programmes

Journal:	*BMJ Open*
Manuscript ID	bmjopen-2023-072234
Article Type:	Protocol
Date Submitted by the Author:	06-Feb-2023

Complete List of Authors:	Roberts, Gareth; Cardiff and Vale NHS Trust,<br>Cahill, Adele; Aneurin Bevan University Health Board<br>Lawthom, Charlotte; Aneurin Bevan University Health Board<br>Price, Martine; Public Health Wales<br>Blyth, Christopher; Aneurin Bevan Health Board<br>Jones, Carys; Bangor University<br>Mc Laughlin, Leah; Bangor University, School of Medical and Health Sciences<br>Noyes, Jane; Bangor University, School of Medical and Health Sciences

Keywords:	Health policy < HEALTH SERVICES ADMINISTRATION & MANAGEMENT, Quality in health care < HEALTH SERVICES ADMINISTRATION & MANAGEMENT, Epilepsy < NEUROLOGY, Parkinson-s disease < NEUROLOGY, Heart failure < CARDIOLOGY, Cataract and refractive surgery < OPHTHALMOLOGY

SCHOLARONE™
Manuscripts

A Protocol for a Realist and Social Return on Investment Evaluation of the use of Patient Reported Outcomes in Four Value Based Healthcare Programmes

Authors

Gareth Roberts,
Value Based Health Care team, Aneurin Bevan University Health Board, Newport, United Kingdom

Adele Cahill
Value Based Health Care team, Aneurin Bevan University Health Board, Newport, United Kingdom

Charlotte Lawthom
Neurology, Aneurin Bevan University Health Board, Newport, United Kingdom

Martine Price
Value Based Health Care team, Aneurin Bevan University Health Board, Newport, United Kingdom

Christopher Blyth
Ophthamology, Aneurin Bevan University Health Board, Newport, United Kingdom

Carys Jones
Centre for Economics and Medicines Evaluation, Bangor University, Bangor, United Kingdom

Leah McLaughlin
School of Medical and Health Sciences, Bangor University, Bangor, United Kingdom

Jane Noyes*
School of Medical and Health Sciences, Bangor University, Bangor, United Kingdom

* Corresponding author:
Jane Noyes
School of Medical and Health Sciences, Fron Heulog, Ffriddoedd Road, Bangor, Gwynedd, LL57 2EF
jane.noyes@bangor.ac.uk
Tel: 01248 388519

Key words
Value in Health, patient outcomes, personalised care, social return, realist evaluation.

Word count: 3944

For peer review only

Abstract (295)

Objectives: There is growing recognition that in order to remain sustainable, the United Kingdom's National Health Service must deliver the best patient outcomes within available resources. This focus on outcomes relative to cost is the basis of Value Based Healthcare (VBHC) and has led to interest in the recording of patient reported outcomes (PROMs) to measure patient perspectives on the impact of a health condition on their lives. The study aim is to understand what works about PROMs collection, for whom, in what contexts, and why in a VBHC context. Additionally, we will assess the social value of integrating PROMs collection into routine care.

Setting: Every Health Board in Wales is now required to collect PROMS as part of routine care. We will evaluate the VBHC programme implemented in a lead Health Board.

Methods and analysis: A 3 stage mixed-methods study comprising of a realist evaluation integrated with Social Return on Investment analysis across four conditions; Parkinson's disease, epilepsy, heart failure and cataract surgery. Workstream 1: Development of logic models, informed by a rapid review, documentary analysis, patient and public involvement (PPI), staff and key stakeholder engagement. Workstream 2: Realist evaluation building on multiple data sources from stages 1 and 3 to test and refine the programme theories that arise from the logic model development. Workstream 3: Social Return on Investment (SROI) analysis using interview data with patients, staff and carers, stakeholder and PPI engagement, anonymised routinely collected data, and questionnaires to populate a model that will explore the social value generated by the implementation of PROMs. Findings across stages will be validated with key stakeholders.

Ethics and dissemination: Approved by Wales Research Ethics Committee #5 (22/WA/0044). Outcomes will be shared with key stakeholders, published in peer-reviewed journals and presented at national and international conferences.

This is an open access article distributed in accordance with the Creative Commons Attribution Non Commercial (CC BY-NC 4.0) license, which permits others to distribute, remix, adapt, build upon this work non-commercially, and license their derivative works on different terms, provided the original work is properly cited, appropriate credit is given, any changes made indicated, and the use is non-commercial

Strengths and limitations of this study

- The Realist evaluation and Social Return on Investment analysis will have a high level of stakeholder involvement, including National Health Service staff, patients, carers and other organisations to provide insights not typically included via alternate study designs and methods.
- Aneurin Bevan University Health Board was one of the first adopters of VBHC in Wales and has been serving as a pilot site for various programmes, including the use of PROMs in direct care. This has led to extensive routinely collected data available for the analyses.

- Developing a deeper understanding of outcomes and their value to real world patient care will have the potential to influence value-based approaches at national and international level.
- The study is limited by time and resources to evaluating four services, nonetheless these have been chosen in order to sample maximum variation i.e., chronic and acute conditions, and conditions affecting a wide age-range.
- COVID-19 will have affected service design and delivery, which will be taken account of in the analyses.

INTRODUCTION

Countries with developed healthcare systems are struggling to meet demand and the needs of their populations (1). Globally there is a growing recognition that healthcare services either have or will soon become unsustainable (2,3). Reasons for this are complex, some include, an increasingly aging, frail and diversifying population(4), increasing co-morbid conditions(5), and lack of patient involvement in interventions designed to address complexity of care and service access (6).

Increasing spending to match demand is no longer a viable solution and accumulated evidence reinforces the view that in order to remain sustainable, the National Health Service (NHS) in the United Kingdom needs to take a different approach (7). One concept that is developing growing recognition, maturity and support as a potential solution is Value Based Healthcare (VBHC) (see glossary of terms; Table 1).

Table 1. Glossary of terms

Acceptability	Explores the perception among stakeholders that the initiatives were agreeable, palatable and/or satisfactory. Usually assessed based on stakeholder knowledge or experience. Various dimensions of initiatives can be considered, such as the content, complexity, and comfort from the perspective of different stakeholders.
Adoption	Otherwise referred to as uptake, refers to the intention, initial decision, or action to implement initiatives.
Appropriateness	Refers to the perceived fit, relevance, or compatibility of initiatives to the implementation setting. Considers the potential resistance to implementation efforts or alignment with care priories from a variety of stakeholder perspectives.
Attribution	An assessment of how much of an outcome can be attributed to the programme or initiative under evaluation
Context	Refers to the literal context in which interventions happen. Contexts can be different for different stakeholders. E.g. 'Busy clinic' would be a clinical context. A single working parent with two small children would be a patient context. Understanding the contexts in which interventions are implemented is vital to understanding (and often predicting) their effectiveness.

For peer review only - http://bmjopen.bmj.com/site/about/guidelines.xhtml

Deadweight	An assessment of the proportion of observed change that stakeholders would experience over the study period, regardless of taking part in Value Based Healthcare (VBHC) Programmes.
Displacement	An assessment of the proportion of potential outcomes that are displaced by other outcomes e.g. implementing a programme to reduce crime in one area could have the unintended effect of displacing crime to a neighbouring region without the crime prevention programme.
Drop-off	An assessment of how long outcomes last into the future.
Feasibility	Explores the extent to which initiatives could be successfully applied within given settings. For example, it may have been considered acceptable and appropriate, but resourcing requirements might have made it unfeasible.
Fidelity	Considered the degree to which the initiatives were implemented as prescribed. Explore the alignment between care received by patients and the model of care developed as part of the initiative. The dimensions of focus include the adherence, quality, component differentiation, exposure to interventions, and patient responsiveness/involvement.
Financial proxy	An estimate of the value of an outcome to the stakeholder experiencing that outcome.
Impact map	A spreadsheet which maps out inputs, outputs, and outcomes in a way which allows quantitative data to be entered to calculate the SROI ratio.
Implementation cost	Conceptualised as the direct cost impact of an implementation attempt. Three components of cost are generally considered: (1) costs of initiative care models; (2) costs of the implementation strategy used; and (3) varying costs of delivery by setting.
Mechanism	Refer to the underlying entities, processes or structures which operate particular contexts e.g. a specialist nurse in busy clinic engaged with PROM development, an online app designed to use 'on the go'/ 'fit into busy lives' are hypothetical examples of mechanisms. There are no limit to the number of mechanisms in a given context.
Middle range theory	A more general explanation drawn from a range of programme theories. A middle range theory could be adapted for use in many different contexts – it is not dependent on a specific disease condition.
Outcome	Refer to the results of an intervention, whether intended or not. E.g. an intended outcomes of routine PROM collection is reduced symptom burden for patients. Hypothetical unintended outcomes could include: more effective use of NHS services; a perception of surplus 'routinely collected patient data' exasperates clinical professionals; or highlights gaps in NHS data analysis and data linking capacity.
Penetration	Otherwise referred to as coverage/saturation, captures the integration of initiatives within settings (e.g. number of initiatives adopted at a site, number of patients utilising a service, number of health professional providing a service).
PROM	Patient Reported Outcome Measures. These are validated questionnaires that patients are requested to complete at various points as part of their routine care. We provide examples in the appendices.
Prudent healthcare	A Welsh Government adopted strategy defined by 4 overall principles: Public and Professionals are equal partners through co-production; care for those with the greatest health need first; do only what is needed and do no harm; reduce inappropriate variation through evidence-based approaches.
Programme theory	An explanation of what a particular programme of work is intended to deliver/improve/change. E.g. Routine PROM collection in cataracts aims to identify patients who will benefit from surgery and use these data to triage patients to more appropriate interventions which may have better patient outcomes.
Realist evaluation	A theory-driven method used to understand the contexts, mechanisms and outcomes underpinning a programme, thereby providing an explanation of 'what works, for whom,

For peer review only

	and in what contexts'. Understanding the way that a programme operates is essential for scaling-up activities.
Retroductive analysis	Retroduction refers to the identification of hidden causal forces that lie behind identified patterns or changes in those patterns. It asks the question: 'why do things appear as they do? Retroduction uses both inductive and deductive logic, as well as insights or hunches. It involves thinking through what causal powers might be at work in producing observed patterns or changes in patterns. It is underpinned by a belief that an understanding of causation cannot be achieved using only observable evidence. Retroductive theorising requires that inquirers use their common sense, intelligence, expertise, and informed imagination to build and test theories about underpinning causal processes.
Social Return on Investment (SROI)	A method used to measure and value outcomes that matter to the people and organisations (stakeholders) who experience them. It incorporates social, economic and environmental value, and produces an SROI ratio which tells us '£x of social value is generated for every £1 invested in the programme under evaluation'.
Social value	A concept which encompasses social, economic and environmental value.
Stakeholder	People or organisations directly affected by the activities of the programme under evaluation
Sustainment	Maintenance of fidelity measurements in an ongoing, stable manner. Emphasises the integration of the initiatives within an organisation's culture. Attaining long-term viability is considered the final stage of diffusion and dissemination within an organisation.
Theory of change	A type of logic model which describes the relationship between inputs, outputs and outcomes/ contexts, mechanisms or outcomes.
Value Based Healthcare (VBHC)	Value Based health Care is an approach which shifts the focus of healthcare onto the outcomes that matter for patients. It is realised when we achieve the best possible health outcomes for our population within available resources. It is a Welsh Government Policy and is linked to all major health and social care policy contexts. One way to support VBHC are PROMs.

Value in health care is realised when we achieve the best possible health outcomes for our population within the resources that we have available (8).

Demonstrating high value care in "real world" settings is challenging since most healthcare systems (including the NHS) have traditionally focused on recording processes (mainly access to care) rather than patient outcomes. The emergence of VBHC as a concept has led to a renewed interest in recording patient reported outcomes (PROMs) as part of routine care (9). PROMs differ from clinician recorded outcomes since they measure patients' perspectives on the impact of disease on their lives. To this end, PROMs have become a central feature of a VBHC approach, with organisations such as International Consortium for Health Outcomes Measurement (ICHOM) recognising the potential benefits for patients from utilising these tools in routine care rather than within the confines of clinical trials (10).

In 2015, the Aneurin Bevan University Health Board (ABUHB; an integrated Healthcare system covering a population of 600,000 in Wales, UK), set up a dedicated VBHC team with the aim of collecting PROMs data across multiple disease areas. The programme has grown exponentially and is now collecting PROMs in over 25 disease areas. In order to support collection at scale, ABUHB partnered with a software supplier (Dr-Doctor) to develop a novel

For peer review only

electronic outcomes capture platform. The platform enables either remote or “in-clinic” collection of electronic PROMs.

One of the key aims of the programme in ABUHB is to use PROMs in direct care as a tool for driving improvement in both patient care and patient outcomes. There are a number of potential mechanisms whereby collecting PROMs may achieve this as described in Table 2.

Table 2 How routine collection of PROMs may lead to improved patient care

**Care domain**	**Potential mechanisms where PROMS may improve care and outcomes**
**Symptom burden**	At the individual patient level, the use of PROMs may empower patients, helping them start conversations with clinicians and focus on symptoms that matter to them. This may improve the detection of problems and support shared clinical decision making.
**Disease trajectory**	In chronic disease settings, temporal changes in symptoms can be plotted over time enabling discussions about disease trajectory and the impact of any treatments. Such data has not traditionally been visible to patients nor clinicians.
**Remote Monitoring**	The use of electronic PROMs lends itself to the development of remote monitoring, enabling virtual clinics and virtual triaging of patients awaiting treatment or review.
**Service improvement**	Feedback of aggregate PROMs data may lead to improved patient care by enabling services to identify which aspects of the service works well and which do not meet patient expectations.
**Benchmarking**	Reporting of case-mix adjusted aggregate PROM data may enable benchmarking with other organisations, thus driving improved care. Aggregate, case-mix adjusted data may also enable shared decision making by giving patients a realistic expectation of what outcomes they may achieve from a given treatment (based on aggregate data derived from similar patient cohorts).

For peer review only

Despite early reported benefits, the PROMs programme within ABUHB has not been formally evaluated. It is important to undertake an evaluation since there are a number of potential barriers which may impact on the goals described above, and thus limit the utility of PROMs. These barriers may include poor completion rates by patients, competing priorities preventing busy clinicians from viewing PROMs, as well as some clinicians lacking the competence or confidence to deal with symptoms uncovered by PROMs. On a service level, resource limitations may mean that appropriate timely referrals and review by other specialities (such as mental health) may not be possible and other solutions such as social prescribing may be required. Another key challenge to PROMs collection is digital exclusion. Technology has transformed healthcare (in particular post COVID); however, many people in the UK cannot access this technology. Those who are excluded are also likely to be disadvantaged socially and economically and hence likely to be the citizens who require healthcare the most (11).

Reviewing the literature for evidence in this area is challenging since most studies of PROMs have related to their use in clinical trials or national audits rather than their use as a tool in routine care. It is clear that the growing use of PROMs in clinical practice has occurred predominantly on the belief that it is intuitively 'a good idea' rather than an approach that is backed by an extensive evidence base (12). The relatively small evidence base that exists for their use in clinical practice is based on studies undertaken almost exclusively in mental health or oncology/palliative care services. Within these services there appears to be conflicting data as to whether PROMs support or constrain patients in sharing or raising issues with clinicians. It would appear that PROMs are useful for patients who preferred not to talk about personal or sensitive issues, helping them to share information (13). It is also clear that some clinicians perceived that standardised PROMs constrained the patient–clinician relationship because they did not capture the complex and dynamic nature of patients' problems (12–18).

Given the dearth of evidence in the literature, the scale and pace at which the Programme in ABUHB is progressing, as well as the ambition of Welsh Government to replicate the work across Wales, it is imperative that the programme undertaken thus far is formally evaluated. In particular we need to fully understand (a) what is working, (b) who it is working for (c) what are the key mechanisms to maximise the use of PROMs to realise VBHC at the levels specified in recent policy contexts.

Aims and objectives

The aim is to undertake a realist evaluation and social return on investment analysis of the electronic collection of PROMS in the first adopter health board in Wales. Our objectives are to:

- Explore whether the PROMs currently collected encapsulate outcomes that matter to patients.
- Evaluate whether PROM collection improves patient care in Parkinson's disease, epilepsy, heart failure and cataract services. Improved patient care might be
  i) more timely,
  ii) closer to home,
  iii) direct referral to relevant health professionals,
  iv) avoid unnecessary hospital visits,
  vi) prevent unplanned admission.
- Identify potential small-scale changes as part of continuous improvement, including service re-design, and improved use of healthcare utilization.
- Measure the social value of integrating PROMs in routine data collection.
- Develop logic models identifying the inputs required for clinicians to utilise PROMs in decision-making, the context, mechanisms of change, and the potential intended/unintended impacts.
- Better understand and develop ways to overcome any barriers associated with electronic PROMs collection, in order to avoid excluding cohorts of people i.e. explore whether the shift to digital collection of PROMs excludes some communities, thus widening healthcare inequalities.

For peer review only

Research questions

1. What works about PROMs collection, for whom, in what contexts, and why in a VBHC context?
2. What is the social return on investment of integrating PROM collection in routine care in a VBHC context?

METHODS

A mixed-methods study comprising of a realist evaluation and social return on investment analysis. The realist and SROI analyses are complementary and will be undertaken in tandem with several key points for integration built into the study design. Combining the approaches will help learn more about what is needed to achieve the goals of VBHC at scale. The study design, overarching processes and integration are illustrated in Fig.1.

Setting

Health services for people with cataracts, epilepsy, heart failure and Parkinson's disease. These are four service areas where PROMs have been routinely collected for a number of years. By choosing four diverse tracer services we will be able to include:

- a surgical intervention (cataracts),
- a chronic disease with a large cohort of young adults (epilepsy), and
- a chronic disease affecting a predominantly elderly and sometimes frail cohort (Parkinson's disease).
- a long-term chronic condition that is most common in older people, but can affect people at any age (heart failure)

The sample across the four tracer services is suitably big enough to address the overall research aims and manageable enough to achieve the objectives within the budget and time constraints. The individual services and their adaption of PROMS to date are described in more detail in Supplementary File 1.

Data collection and analysis

Work stream 1 (WS1)*: Rapid review, documentary analysis and stakeholder engagement to develop logic models and refine programme theories for the Realist evaluation and Social Return on Investment analysis*

Rapid review: Primary searches will be conducted in CINAHL, Cochrane CENTRAL, EMBASE, MEDLINE, PsycINFO, PubMed and Web of Science, and we will include relevant reports found via key word searches on Google Scholar. We will liaise with partners in ABUHB to identify additional relevant literature. To minimise the risk of bias, the quality of papers will be assessed using a standardised checklist such as the AACODS checklist for grey literature (19) or the CASP checklists for primary studies (20). Framework synthesis methods will be used to synthesise findings.

For peer review only

Documentary analysis: we will undertake content analysis (21) using NVIVO of the VBHC service specification, change and implementation documents (e.g. PROMS data, administrative patient data, audits, evaluation surveys, clinical processes and outcomes) for Parkinson's disease, epilepsy, heart failure and cataract surgery services. We will identify the key elements needed to develop initial programme theories and logic, and build an understanding of implementation processes for each tracer service

Engagement with key stakeholders: such as VBHC steering group members, programme designers, clinical implementers and PPI representatives will also inform the development of the initial logic models and programme theories.

Through undertaking WS1, we will uncover the underlying system dynamics (relationships between agents, their attributes and rules of behaviour, network structure, feedback loops) that influence implementation and bring about outcomes. The initial logic models and programme theories will be further developed as the study progresses and serve as the theoretical framework for the study.

Workstream 2 (WS2): Realist evaluation

We will follow the methods of Pawson (22) and Rycroft-Malone (23) and focus will shift from developing initial theories in WS1 to testing theories and refining the narrative that best explains the implementation of VBHC and observed outcomes for people in the tracer services. We will explain the circumstances (context) and mechanisms that drive outcomes (CMOs) in each tracer service. CMOs will be mapped against the predetermined programme theory to determine what is and is not working and the mechanism of action to achieving the observed outcomes (positive and negative).

We will continue to engage with key stakeholder groups and PPIs.

We will conduct realist exploratory semi-structured interviews with key people in implementing PROMs such as VBHC network staff, local healthcare professionals in the tracer services, patients and carers, and combine with quantitative process data. PROMS outcome data and how they are valued will be used to test and refine CMOs constructed. We will also ask patients for their NHS number to cross check against anonymized PROMS data to identify if they have received and returned PROMS as anticipated. Interviews will be recorded, transcribed and uploaded into NVIVO for analysis.

Consistent with realist approaches, analysis will be retroductive in that it will oscillate between an inductive and deductive logic to multiple data sources as well as incorporating the researchers' own insights and PPI /stakeholder views of what causes something to work (causation) for the programme theories. We will read through and systematically analyse each interview transcript or fieldnote and code data of interest that helps explain why and how something is working or not in a specific context. Each stage of the iterative realist analysis will become progressively focused on CMO configurations. Overall, data will be analysed within data sources (documents, interviews, process and outcomes, and service evaluation data), and with key stakeholders, PPIs and steering group members over time, and then explanations will be developed across the data, with attention to the realist task of

For peer review only - http://bmjopen.bmj.com/site/about/guidelines.xhtml

uncovering contingencies and conditions i.e. the relationships between factors that explain ABUHB's approach to implementation of PROMs, and the conditions in which they operate. We will then revisit the programme theories and refine these in light of CMO data to confirm or disconfirm our CMOs, ensuring a diversity of data sources and another level of clarification. We will map the actual process of implementation (work as done) compared to how it was planned (work as imagined). We will also translate CMOs into generalisable theoretical models (middle-range theories) for implementing large-system, VBHC programmes. The process will begin by presenting and defining CMOs with key stakeholders. Further testing will be undertaken by explicitly seeking disconfirming or contradictory data and considering other Interpretations that might account for the same findings.

We will review the realist findings for the tracer services (including programme theories and CMO configurations) 'vertically' to identify common thematic elements according to context, mechanisms and outcomes. Data will also be analysed across each service 'horizontally' to uncover potential generative causal patterns between mechanisms and outcomes. This process will potentially translate the specifics of implementing VBHC in the tracer services to more analytically driven generalisable theories for scaling up the benefits from these care delivery models to achieve large system transformation across Health Boards in Wales and beyond.

Workstream 3 (WS3): Social Return on Investment (SROI) analysis
We will develop an overall programme-level theory of change to establish how inputs (e.g. costs, staffing) are converted into outputs (e.g. numbers of patients seen), and subsequently into outcomes that matter to stakeholders affected by the programme (e.g. improved mental health). The social value generated by these outcomes is then estimated in a similar way to cost-benefit analysis.

SROI analysis will be operationalised through the stages outlined in the Guide to Social Return on Investment analysis (24):

1. **Establishing scope and identifying stakeholders (carried out in WS1)**
The scope of the study is to evaluate the social value generated by the use of PROMS in the VBHC programmes for the four services. Stakeholder involvement is critical to both the design and conduct of the study; particularly in relation to the development of programme theories. To identify stakeholders, we will list all potential groups who might affect or be affected by the activities of the programmes listed above.

2. **Mapping outcomes**
The next step is to identify the potential outcomes of each programme (positive or negative, intentional or unintentional). Informed by the documentary analysis and logic models developed in WS1, an impact map will be created for each service to explain the relationship between programme inputs, outputs and outcomes for each stakeholder group, and how these outcomes can generate value.

3. **Evidencing outcomes and giving them a value**
Longitudinal data on PROMs and clinical outcomes will be extracted at an anonymized, aggregated level allowing us to evaluate the relationship between PROM collection and the

For peer review only - http://bmjopen.bmj.com/site/about/guidelines.xhtml

outcomes experienced by patients. Data will be aggregated by age, gender, and clinical severity. A benefit of using routinely collected data is that it facilitates access to a larger cohort than would be possible with prospective data collection, thus reducing research time and costs.

It will be important to define what will be considered a material change for each of the outcomes being measured. For example, increased physical activity may be an outcome that patients experience as a result of their treatment; however, a *material* change in physical activity could be defined as a patient crossing the threshold from not meeting, to now meeting, the NHS recommendation of undertaking 150+ minutes of moderate intensity activity per week. Once the magnitude of change experienced by each stakeholder group has been identified through a combination of routinely collected data; the next step of the SROI analysis involves assigning a monetary value to the outcomes experienced by stakeholders using a financial proxy.

Outcomes occurring beyond one year will be discounted by 3.5% per annum to minimise the risk of over-claiming the amount of social value generated by the programmes.

The cost of inputs required to deliver PROMs in the tracer services will be identified in consultation with VBHC leads at ABUHB.

4. **Establishing impact**

Establishing impact is necessary to reduce bias and the risk of over-claiming the benefits of the programme. We will include model parameters to take into account deadweight (the proportion of observed outcomes that would have happened to stakeholders without PROMS); displacement (the proportion of outcomes that have been displaced from one sector to another); attribution (the amount of observed outcomes that can be direct attributed to the programme), and drop-off (the length of time that outcomes last for stakeholders). The model inputs for these variables may vary between stakeholder groups, so values will be established through stakeholder interviews and routinely collected data.

5. **Calculating the SROI**

Microsoft Excel will be used to create a model for running the SROI analysis. The model variables will be identified through the logic models developed in WS1 and further evolved in WS2. The parameters for attribution, deadweight, displacement, and drop-off will be derived from analysis of routinely collected data and varied in a range of one-way sensitivity analyses. The model will be populated with data obtained through the extraction of routinely collected data. The SROI ratio is calculated by dividing the total value of outcomes by the total value of inputs across all stakeholders. The resulting ratio is the amount of social value generated for every £1 invested in the programmes. In addition to calculating the base case scenario we will perform a range of sensitivity analyses to explore how the SROI ratio would be affected if various input parameters were changed, different financial proxies were used, and varying levels of outcomes were achieved to those observed in the base case. A checklist for quality assessment in SROI analysis (25) will be used as a framework to guide the reporting of the findings.

Identification of routinely collected data for sharing in WS2 and 3

For peer review only - http://bmjopen.bmj.com/site/about/guidelines.xhtml

We will identify what routinely collected data of specific interest related to VBHC and PROM implementation in the four services (e.g. PROMS data, administrative patient data, audits, evaluation surveys, clinical processes and outcomes) could be used in the realist evaluation and SROI. We will then develop a process to securely share these anonymized data between ABUHB and the research team.

Sampling

There is no minimum sample size for realist evaluation or SROI analysis as these types of studies are primarily used to develop explanatory theory rather than to detect statistical significance. Therefore we will interview a minimum representative sample of 15 patients and carers (separately or together) from each of the four tracer services in Epilepsy, Parkinson's disease, Cataract Surgery and Heart Failure (min 45 interviews), and up to 10 staff members responsible for implementing PROMs in routine practice from each of the tracer services.

Recruitment strategy

Patients, carers and consultees

We will purposively select patients according to their age, gender, ethnicity, service and whether they completed a PROM or not. We may add additional attributes and classifications to the sampling frame as appropriate. Patients will be contacted by ABUHB staff in the first instance and carers will be recruited through the patients. Routes to recruitment include:

- ABUHB will send out an invitation and study information via their internal PROMs platform.
- Routine appointments or via email, or post.

Staff members can support potential participants to complete and return a consent to contact form or the potential participants can self-complete and return in pre-paid envelopes. For participants lacking mental capacity a consultee will be appointed. Once a consent to contact form is received the research team will follow up to organize an interview.

Staff

Staff involved in the VBHC programme generally or in the four services specifically will initially be approached by ABUHB staff via meetings, direct mails and face to face contact. Purposive sampling and snowballing will be used to identify respondents best placed to provide information on PROM implementation and outcomes.

Inclusion Criteria

- Stakeholders involved with the implementation of PROMs and VBHC Programmes in ABUHB and where relevant other health boards in Wales.
- All professionals in ABUHB involved in the routine collection of PROMs in the tracer services.

- Patients aged over 18 in the identified services.
- Carers over 18 linked to a patient who is receiving care in the identified tracer services.
- Identified consultees for people who lack capacity to consent.

Exclusion Criteria

- People under 18.
- People who do not have the capacity to consent to take part in the research and for whom a consultee is not available.

Patient and public involvement (PPI)

The study was developed alongside a wide range of PPI stakeholders involved in ABUHB, the VBHC programme, third sector, and specific individuals and groups representing the tracer services. Going forward, partner organisations will have a key role in the interpretation and application of findings ensuring that outcomes have relevance and are accessible to specific needs and circumstances of underrepresented or especially vulnerable groups. This will be important as we know that PROMs have the capacity to help those with the greatest need first, but these individuals may encounter the most barriers to complete a PROM for example, disability, health literacy or socioeconomic status. Our partners can help advise on the ways identified Context Mechanism Outcomes chains may need to be modified or adapted to account for the needs of these patients in Wales.

Figure 2 maps and helps to visualise opportunities for PPI involvement and engagement with the multiple groups already established as part of the overall VBHC Programme and how we imagine PPI to help support adoption and scale-up of the study outcomes. We will follow the UK standards for patient and public involvement throughout (26).

Ethical issues

The study has full approval from Health and Care Research Wales Research Ethics Committee #5 (22.WA/0044). Box.1 outlines the ethical issues we have considered in designing this study

Box.1 Key ethical issues relevant to this study

Capacity to consent and personal consultees

Informed consent will be sought from all participants. Where there are concerns over capacity to consent, thorough discussions will be undertaken within the research team and a final decision will be made by the lead researcher regarding including participants who lack capacity (either temporarily or permanently). Where participants are found to be lacking capacity, a personal consultee may be appointed.

Large print versions of documents such as study information sheets and consent forms will be available for participants with a visual impairment (e.g. those attending the cataracts service). We will also provide audio recordings and explanations of the study and consent processes.

For peer review only

Risk of distress to participants, and pathways to referral if concerns are raised

The research team acknowledge that taking part in this study will mean that participants will have to think about and reflect upon their experiences, which may raise some concerns for individuals. Should participants be concerned by the subject matter or by any issues raised, staff participants will be signposted to consult with their line manager or occupational health department for support and patient participants will be signposted to the relevant health professional. The research team have detailed distress protocols to follow in these situations and are experienced in collecting sensitive data around personal experiences of healthcare support and living with long term health conditions.

Potential identification of poor clinical practice and unmet need

Researchers are experienced healthcare scientists. Standard data protection and confidentiality protocols will be followed. We will only deviate from these if we recognize a clear and immediate risk to the health and wellbeing of participants. We will partner with wider social support services and signpost to these as part of the disengagement process we may also ask participants if they would like us to send a letter to the relevant healthcare professional alerting them to the fact that they have taken part in the study.

Maintaining confidentiality of professional participants

Professionals will be provided with an information sheet and consent form explaining that their participation is anonymous. If there is a risk of identification e.g. small team, recognizable quotes etc. we will group participants under 'professionals' and work to remove any content that may lead back to any one individual. Healthcare professionals working on the study are aware that there is no intention to identify participants at an individual level and rather to present information in general terms to facilitate learning and professional practice.

Dissemination and impact

As a co-productive study involving patients, clinicians, third sector partners and academics, findings will be shared on a continual basis. Outputs will be disseminated widely through patient and clinical networks, policy and academic routes. Consistent with realist and SROI methodology, we will disseminate findings to stakeholders as they become available through presentations, meetings and events. We will host an interim event with representatives from the multiple stakeholder groups to present initial outcomes and gain feedback and direction for the remainder of the study.

We will produce a lay report to include infographics and visuals supporting wider understanding of the VBHC strategic ambition.

We speculate that the outputs of the study will include:

- A deeper understanding of the impacts of the switch to online PROMs specific to the Welsh population.

For peer review only

- A series of programme implementation theories explaining what is needed to realise VBHC at scale.
- A final report which will include examples of the contextual barriers and facilitators that promote the uptake of PROMS, thus enhancing understanding of how to implement them successfully in other health boards.
- An understanding of how social value is generated to different stakeholders, leading to a greater understanding of how best to optimise services to reduce inefficiencies, improve outcomes and maximise value.
- An upskilled, adaptive and responsive workforce with greater understanding of the impact and value of incorporating PROMs data in their decision-making; a workforce equipped to handle the ever-evolving role of healthcare professionals.

List of Tables, figures and appendices

Table 1 Glossary of terms
Table 2 Ways PROMS may improve care and outcomes

Figure 1. Outline of realist evaluation with SROI integration and explanation of processes
Figure 2. Map of PPI partners and their proposed ongoing involvement throughout the study and their capacity to influence scale up

Supplementary File 1. Individual services and their adaption of PROMS to date

References

1. Healthcare costs unsustainable in advanced economies without reform - OECD [Internet]. [cited 2022 Dec 11]. Available from: https://www.oecd.org/health/healthcarecostsunsustainableinadvancedeconomieswithoutreform.htm
2. The unsustainable trajectory of global healthcare and ways forward - Copenhagen Economics [Internet]. [cited 2022 Dec 11]. Available from: https://copenhageneconomics.com/publication/the-unsustainable-trajectory-of-global-healthcare-and-ways-forward/
3. Estimating the financial cost of chronic kidney disease to the NHS in England | Nephrology Dialysis Transplantation | Oxford Academic [Internet]. [cited 2020 Jun 12]. Available from: https://academic.oup.com/ndt/article/27/suppl_3/iii73/1822662?related-urls=yes&legid=ndt;gfs269v3
4. Care Quality Commission. 2021 [cited 2022 Dec 11]; Available from: www.gov.uk/official-documents.
5. Raymond A, Bazeer N, Barclay C, Krelle H, Idriss O, Tallack C, et al. Our ageing population: how ageing affects health and care need in England. 2021 Dec 13 [cited 2022 Dec 11]; Available from: https://www.health.org.uk/publications/our-ageing-population
6. Mclaughlin L. Assessing the efficacy of co-production to better understand the barriers to achieving sustainability in NHS chronic kidney services and create alternate pathways. BMJ Open. 2021;
7. Crisp N. What would a sustainable health and care system look like? BMJ [Internet]. 2017 Sep 1 [cited 2020 Aug 9];358. Available from: http://www.bmj.com/permissionsSubscribe:http://www.bmj.com/subscribeBMJ2017;358:j3895doi:10.1136/bmj.j3895
8. Braithwaite J, Glasziou P, Westbrook J. The three numbers you need to know about healthcare: The 60-30-10 Challenge. Vol. 18, BMC Medicine. 2020.
9. Withers K, Palmer R, Lewis S, Carolan-Rees G. First steps in PROMs and PREMs collection in Wales as part of the prudent and value-based healthcare agenda. Qual Life Res. 2020;
10. Kelley TA. International Consortium for Health Outcomes Measurement (ICHOM). Trials. 2015;16(S3).

For peer review only

11. Marmot M. An inverse care law for our time. BMJ [Internet]. 2018 [cited 2022 Dec 11];362. Available from: https://pubmed.ncbi.nlm.nih.gov/30065009/
12. Greenhalgh J, Dalkin S, Gooding K, Gibbons E, Wright J, Meads D, et al. Functionality and feedback: a realist synthesis of the collation, interpretation and utilisation of patient-reported outcome measures data to improve patient care. Heal Serv Deliv Res. 2017;5(2).
13. Hall CL, Taylor J, Moldavsky M, Marriott M, Pass S, Newell K, et al. A qualitative process evaluation of electronic session-by-session outcome measurement in child and adolescent mental health services. BMC Psychiatry. 2014;14(1).
14. Annells M, Koch T. 'The real stuff: Implications for nursing of assessing and measuring a terminally ill person's quality of life. J Clin Nurs. 2001;10(6).
15. Hagelin CL, Wengtröm Y, Tishelman C, Fürst VJ. Nurses' experiences of Clinical use of a quality of life instrument in palliative care. Contemp Nurse. 2007;27(1).
16. Mitchell C, Dwyer R, Hagan T, Mathers N. Impact of the QOF and the NICE guideline in the diagnosis and management of depression: A qualitative study. Br J Gen Pract. 2011;61(586).
17. Fagerlind H, Lindblad ÅK, Bergström I, Nilsson M, Nauclér G, Glimelius B, et al. Patient-physician communication during oncology consultations. Psychooncology. 2008;17(10).
18. Pettersson A, Björkelund C, Petersson EL. To score or not to score: A qualitative study on GPs views on the use of instruments for depression. Fam Pract. 2014;31(2).
19. Tyndall J. AACODS checklist for grey literature. 2010.
20. CASP. CASP checklist for systematic reviews. 2018.
21. Hsieh HF, Shannon SE. Three approaches to qualitative content analysis. Qual Health Res [Internet]. 2005 Nov 1 [cited 2022 Jul 26];15(9):1277–88. Available from: https://journals.sagepub.com/doi/10.1177/1049732305276687
22. Pawson R, Tilley N. Realistic Evaluation. London: Sage Publications; 1997.
23. Rycroft-Malone J, McCormack B, Hutchinson AM, DeCorby K, Bucknall TK, Kent B, et al. Realist synthesis: illustrating the method for implementation research. Implement Sci. 2012;7(1).
24. Nicholls J, Lawlor E, Neitzert E GT. A Guide to Social Return on Investment. London; 2012.
25. Hutchinson CL, Berndt A, Gilbert-Hunt S, George S, Ratcliffe J. Valuing the impact of health and social care programmes using social return on investment analysis: How have academics advanced the methodology? A protocol for a systematic review of peer-reviewed literature. BMJ Open. 2018;8(12).
26. UK Standards for Public Involvement [Internet]. [cited 2021 Sep 6]. Available from: https://sites.google.com/nihr.ac.uk/pi-standards/home

Authors' contributions

The study concept and design was conceived by GR, AC and JN. CL, MP, CB, CJ and LM assisted in refining the study questionnaires and study design. GR prepared the first draft of the manuscript. All authors critically revised the manuscript and approved the submitted version.

Funding statement

This work was funded by Health and Care Research Wales (HCRW). The views expressed are those of the authors and not necessarily those of NHS Wales and HCRW

Competing interests statement

GR, AC, MP, CL and CB are employed by the Health Board whose programme is under evaluation.

Acknowledgements

Thank you to Angie Pullen Director of Epilepsy Services, Ana Palazon Country Director Wales, Parkinson’s UK Cymru and Chair of the Wales Neurological Alliance, Owen Williams Chair of the Wales Council for the Blind. Thank you also to Roon Adams, Race Equality First; Lorraine Morgan, Action for Elders; Dawn Owen, Carers Wales and Chris Hodson Gwent Citizens Panel. Key representatives from these groups have been involved in selecting the research questions, the study design (including scope and sampling), the realist model and examples of Context, Mechanism, Outcome chains of evidence from the tracer services, reviewing recruitment documents and supporting the ethics application.

Word count: 3944

For peer review only

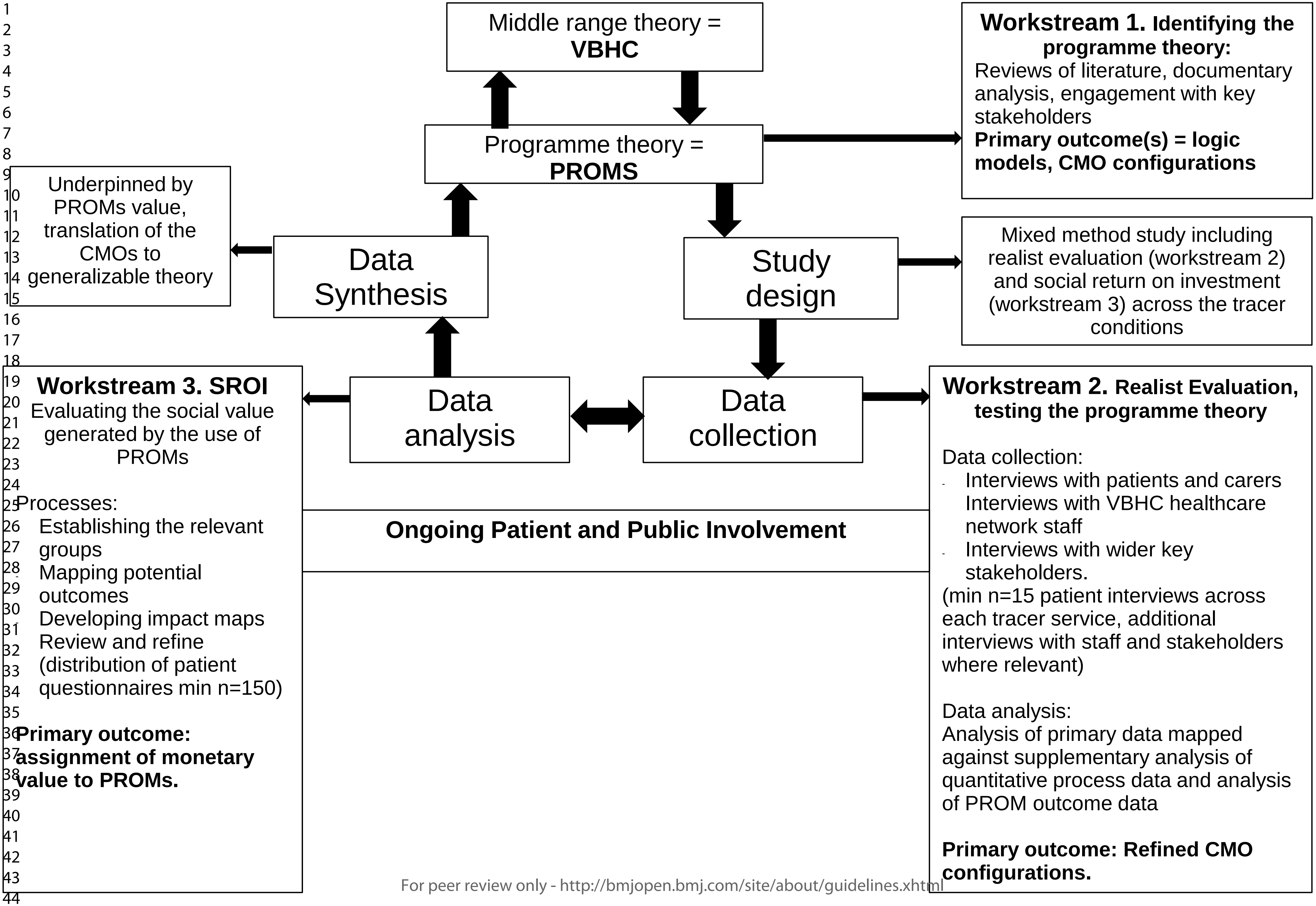

For peer review only - http://bmjopen.bmj.com/site/about/guidelines.xhtml

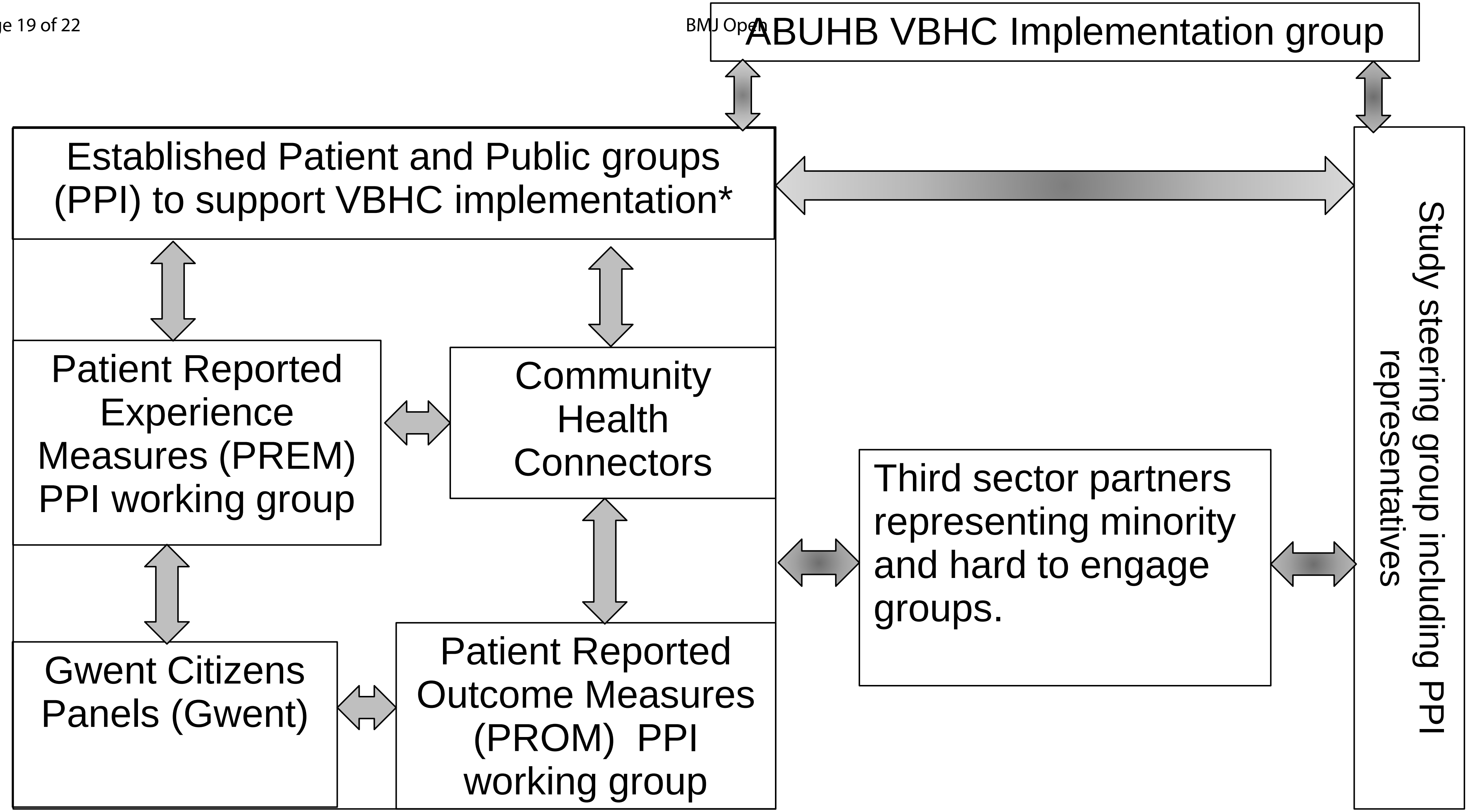

*Every health board in Wales will have PPI structures and working groups

Appendix. 1 Further explanation of the four tracer conditions and their implementation of PROMS to date

Description of the overall VBHC Programme in the adopter Health Board, and the tracer services

Initiative	Patient population	Description
Adopter health board (Aneurin Bevan UHB) implements VBHC from 2015 to present. 26 disease conditions included to date.	Health board serves approx. 639,000 patients; approx. 21% of total Welsh Population	Value Based Health Care: puts a focus on the value of outcomes achieved for patients. Value in health care is realised when we achieve the best possible health outcomes for our population within the resources that we have available.
**Tracer services**		
Epilepsy routine collection of PROMs 2017 to present	588 PROMs completed with an average completion rate of 51%	Identifying, quantifying and managing the burden of mood disorder. Identify relationship between patients mood, seizure frequency and medication to inform future treatment options
Parkinson's disease routine collection of PROMs 2017 to present.	840 PROMs completed with an average completion rate of 42%	Using PROMs to help deliver a more efficient and effective service based on disease severity and patient needs.
Heart Failure routine collection of PROMs 2018 to present	927 PROMs completed with an average completion rate of 85%	Using PROMs to reorganise the service more efficiently to reduce nurses' caseloads, reduce waiting times for patients and to focus treatment on patients' needs
Cataract routine collection of PROMs 2017 to present	3600 PROMs completed with an average completion rate of 88%	Using PROMs to redesign the referral and triage process, freeing up capacity and improving outcome by treating according to need rather than waiting times.

For peer review only

More detailed explanation of PROM integration to date

Tracer service 1: Cataracts

Cataract procedures are the most common operation performed in the UK, with more than 300,000 procedures carried out each year. The aim of cataract surgery is to improve quality of vision for patients and allow patients the ability to undertake everyday tasks. ABUHB receives 6000 cataracts referrals and performs more than 3,000 cataract operations every year. Demand for cataract surgery has exceeded capacity. Within ABUHB, a pilot study identified potential variation in pathways and outcomes. The VBHC team worked with the ophthalmology directorate to set up systematic collection of both clinical outcomes and PROMs. Visual acuity (ability to read letters of decreasing size) from a set distance was used as the clinical outcome. A cataract questionnaire consisting of 9 questions (CatQuest-9SF tool) was used as the validated PROM. The Catquest questionnaire explores visual impairment from a patient's perspective by asking questions about the ability to recognise faces, read newspapers or undertake hobbies. Preliminary data analysis of the first 600 cases suggested that (based on PROM scores) up to 20% of patients did not actually seem to benefit

Appendix. 1 Further explanation of the four tracer conditions and their implementation of PROMS to date

from surgery i.e. their visual disability did not subjectively improve after cataract surgery. Within this cohort, pre-operative PROMs scores seemed a good predictor of who would benefit the most from surgery. This has led the service to consider how pre-operative PROMs could support triage to appropriate services and inform shared decisions about surgery. We also need to better understand from the perspective of the patient and their carers what outcomes are of importance to them and whether the appropriate outcomes are being measured.

Tracer service 2: Epilepsy

The adult Epilepsy Service in ABUHB serves a population of over 6000 people with epilepsy and provides an open access service so that patients with this unpredictable condition can receive timely and responsive care. Patients can access this service via telephone or email and most patients are responded to within one working day. The service has long been aware of the rates of unmet mental health needs in the epilepsy population. There is clear data linking all forms of poor mental health with increase in seizures [23], [24]. The implementation of VBHC in ABUHB was undertaken with the aim of using PROMs to better identify and quantify the extent of anxiety and depression in people with epilepsy. Additionally, the team wanted to improve the management of mental health at individual patient level and also to track changes over time to assess whether epilepsy medication was positively or negatively affecting mood.

The Epilepsy PROM comprises questions on seizure frequency and epilepsy drugs, plus mood scores and a patient global impression change. Initial feedback from people with epilepsy revealed that the patients were overall, very happy to be asked about mood but expected feedback to them about that data and about what should happen next. The high rate of markers for suicidality uncovered by utilising PROMS scores in people with epilepsy was a deeply troubling (but potentially lifesaving) finding, but it is unclear at this stage if patient outcomes have improved.

The COVID pandemic forced the service to move to remote consultation in up to 95% of cases. COVID was also associated with an increasing demand for epilepsy services with the vast majority of people (with epilepsy) suffering with poor mood.

In terms of acting on PROMs data, in the past year the service has made:

- 18 referrals to the Mental Health team,
- 34 referrals to the Online Cognitive Behavioural Therapy (CBT) system (Silver cloud)
- 112 have been referred to online and application based mindfulness tools.
- A further 28 people have been commenced on antidepressant therapy.

PROMs are subsequently used to assess the benefits of these interventions. Please see Appendix 4 for case studies. At a local level the potential benefit to patients is clear in addition to potentially realizing the aims of VBHC, but there has been no formal evaluation to determine the mechanisms or patient outcomes and this needs addressing.

Tracer service 3: Parkinson's Disease (PD)

Parkinson's disease is a progressive nervous system disorder that affects movement. Tremors are common, but the disorder also commonly causes stiffness or slowing of movement. Within ABUHB, PROMs have been routinely collected in the PD clinic for a number of years, indeed PD was the first service to start collecting PROMs in ABUHB. The PROMs were collected in person at attendance in clinic with the PD specialist nurse, who had sight of the scores before seeing them. As well as the individual benefit of capturing all of the patient concerns, the PROMs data enables categorisation of people with PD into newly diagnosed, moderately affected and complex PD. It became clear that these patient groups had different health and social care needs with the more complex patients requiring a number of additional services

For peer review only

Appendix. 1 Further explanation of the four tracer conditions and their implementation of PROMS to date

such as physiotherapy and speech and language therapy. This led the service to begin overhauling the traditional clinic structure so that more complex patients could be stratified into a dedicated multidisciplinary clinics where their needs could all be addressed. Unfortunately, this development was paused during the COVID pandemic therapy which, as in other areas led to a wholescale switch to remote (rather than in-clinic) collection of PROMs. Of note, in spite of concerns that a more elderly cohort may not embrace remote collection, completion rates have been surprisingly high at 54%. A largescale evaluation is now needed to explore these issues and service overhaul in depth to see if patient outcomes have improved.

Tracer serviced 4: Heart Failure

Heart failure is a long-term condition that tends to get gradually worse over time. It cannot usually be cured, but the symptoms can often be controlled for many years. PROMs were introduced in the Heart Failure service in 2018, because the service was overwhelmed with patients and unable to meet demand. Patients were seen in an untimely manner and not necessarily in relation to their symptoms. The service introduced PROMs in an attempt to be more patient-focused. Previously, PROMs were applied at one hospital site using non-clinical staff to administer, but for a variety of reasons this was not sustainable. However, since the introduction of PROMS in the service, the service has developed a broader picture of care at a patient-level, of service improvement and of understanding the population needs of the service.

For peer review only

A Protocol for a Realist and Social Return on Investment Evaluation of the use of Patient Reported Outcomes in Four Value Based Healthcare Programmes

Journal:	*BMJ Open*
Manuscript ID	bmjopen-2023-072234.R1
Article Type:	Protocol
Date Submitted by the Author:	23-Feb-2023
Complete List of Authors:	Roberts, Gareth; Cardiff and Vale NHS Trust,<br>Cahill, Adele; Aneurin Bevan University Health Board<br>Lawthom, Charlotte; Aneurin Bevan University Health Board<br>Price, Martine; Public Health Wales<br>Blyth, Christopher; Aneurin Bevan Health Board<br>Jones, Carys; Bangor University<br>Mc Laughlin, Leah; Bangor University, School of Medical and Health Sciences<br>Noyes, Jane; Bangor University, School of Medical and Health Sciences
**Primary Subject Heading**:	Health services research
Secondary Subject Heading:	Evidence based practice, Health informatics, Health policy, Health economics, Public health
Keywords:	Health policy < HEALTH SERVICES ADMINISTRATION & MANAGEMENT, Quality in health care < HEALTH SERVICES ADMINISTRATION & MANAGEMENT, Epilepsy < NEUROLOGY, Parkinson-s disease < NEUROLOGY, Heart failure < CARDIOLOGY, Cataract and refractive surgery < OPHTHALMOLOGY

A Protocol for a Realist and Social Return on Investment Evaluation of the use of Patient Reported Outcomes in Four Value Based Healthcare Programmes

Authors

Gareth Roberts,
Value Based Health Care team, Aneurin Bevan University Health Board, Newport, United Kingdom

Adele Cahill
Value Based Health Care team, Aneurin Bevan University Health Board, Newport, United Kingdom

Charlotte Lawthom
Neurology, Aneurin Bevan University Health Board, Newport, United Kingdom

Martine Price
Value Based Health Care team, Aneurin Bevan University Health Board, Newport, United Kingdom

Christopher Blyth
Ophthamology, Aneurin Bevan University Health Board, Newport, United Kingdom

Carys Jones
Centre for Economics and Medicines Evaluation, Bangor University, Bangor, United Kingdom

Leah McLaughlin
School of Medical and Health Sciences, Bangor University, Bangor, United Kingdom

Jane Noyes*
School of Medical and Health Sciences, Bangor University, Bangor, United Kingdom

* Corresponding author:
Jane Noyes
School of Medical and Health Sciences, Fron Heulog, Ffriddoedd Road, Bangor, Gwynedd, LL57 2EF
jane.noyes@bangor.ac.uk
Tel: 01248 388519

Key words
Value in Health, patient outcomes, personalised care, social return, realist evaluation.

Word count: 3944

For peer review only

Abstract (295)

Objectives: There is growing recognition that in order to remain sustainable, the United Kingdom's National Health Service must deliver the best patient outcomes within available resources. This focus on outcomes relative to cost is the basis of Value Based Healthcare (VBHC) and has led to interest in the recording of patient reported outcomes (PROMs) to measure patient perspectives on the impact of a health condition on their lives. The study aim is to understand what works about PROMs collection, for whom, in what contexts, and why in a VBHC context. Additionally, we will assess the social value of integrating PROMs collection into routine care.

Setting: Every Health Board in Wales is now required to collect PROMS as part of routine care. We will evaluate the VBHC programme implemented in a lead Health Board.

Methods and analysis: A 3 stage mixed-methods study comprising of a realist evaluation integrated with Social Return on Investment analysis across four conditions; Parkinson's disease, epilepsy, heart failure and cataract surgery. Workstream 1: Development of logic models, informed by a rapid review, documentary analysis, patient and public involvement (PPI), staff and key stakeholder engagement. Workstream 2: Realist evaluation building on multiple data sources from stages 1 and 3 to test and refine the programme theories that arise from the logic model development. Workstream 3: Social Return on Investment (SROI) analysis using interview data with patients, staff and carers, stakeholder and PPI engagement, anonymised routinely collected data, and questionnaires to populate a model that will explore the social value generated by the implementation of PROMs. Findings across stages will be validated with key stakeholders.

Ethics and dissemination: Approved by Wales Research Ethics Committee #5 (22/WA/0044). Outcomes will be shared with key stakeholders, published in peer-reviewed journals and presented at national and international conferences.

This is an open access article distributed in accordance with the Creative Commons Attribution Non Commercial (CC BY-NC 4.0) license, which permits others to distribute, remix, adapt, build upon this work non-commercially, and license their derivative works on different terms, provided the original work is properly cited, appropriate credit is given, any changes made indicated, and the use is non-commercial

Strengths and limitations of this study

- Realist evaluation and Social Return on Investment methods are ideally suited to exploring what happened when patient reported outcome measures were implemented into routine practice as part of the Value-Based Healthcare Programme.
- The Realist evaluation and Social Return on Investment analysis will have a high level of stakeholder involvement, including National Health Service staff, patients, carers and other organisations to provide insights not typically included via alternate study designs and methods.
- Aneurin Bevan University Health Board was one of the first adopters of VBHC in Wales and has a large repository of routinely collected data available for the analyses.

- The study is limited by time and resources to evaluating four services, nonetheless these have been chosen in order to sample maximum variation i.e., chronic and acute conditions, and conditions affecting a wide age-range.

INTRODUCTION

Countries with developed healthcare systems are struggling to meet demand and the needs of their populations (1). Globally there is a growing recognition that healthcare services either have or will soon become unsustainable (2,3). Reasons for this are complex, some include, an increasingly aging, frail and diversifying population(4), increasing co-morbid conditions(5), and lack of patient involvement in interventions designed to address complexity of care and service access (6).

Increasing spending to match demand is no longer a viable solution and accumulated evidence reinforces the view that in order to remain sustainable, the National Health Service (NHS) in the United Kingdom needs to take a different approach (7). One concept that is developing growing recognition, maturity and support as a potential solution is Value Based Healthcare (VBHC) (see glossary of terms; Table 1).

Table 1. Glossary of terms	
Acceptability	Explores the perception among stakeholders that the initiatives were agreeable, palatable and/or satisfactory. Usually assessed based on stakeholder knowledge or experience. Various dimensions of initiatives can be considered, such as the content, complexity, and comfort from the perspective of different stakeholders.
Adoption	Otherwise referred to as uptake, refers to the intention, initial decision, or action to implement initiatives.
Appropriateness	Refers to the perceived fit, relevance, or compatibility of initiatives to the implementation setting. Considers the potential resistance to implementation efforts or alignment with care priories from a variety of stakeholder perspectives.
Attribution	An assessment of how much of an outcome can be attributed to the programme or initiative under evaluation
Context	Refers to the literal context in which interventions happen. Contexts can be different for different stakeholders. E.g. 'Busy clinic' would be a clinical context. A single working parent with two small children would be a patient context. Understanding the contexts in which interventions are implemented is vital to understanding (and often predicting) their effectiveness.
Deadweight	An assessment of the proportion of observed change that stakeholders would experience over the study period, regardless of taking part in Value Based Healthcare (VBHC) Programmes.
Displacement	An assessment of the proportion of potential outcomes that are displaced by other outcomes e.g. implementing a programme to reduce crime in one area could have the unintended effect of displacing crime to a neighbouring region without the crime prevention programme.
Drop-off	An assessment of how long outcomes last into the future.
Feasibility	Explores the extent to which initiatives could be successfully applied within given settings. For example, it may have been considered acceptable and appropriate, but resourcing requirements might have made it unfeasible.

For peer review only

Fidelity	Considered the degree to which the initiatives were implemented as prescribed. Explore the alignment between care received by patients and the model of care developed as part of the initiative. The dimensions of focus include the adherence, quality, component differentiation, exposure to interventions, and patient responsiveness/involvement.
Financial proxy	An estimate of the value of an outcome to the stakeholder experiencing that outcome.
Impact map	A spreadsheet which maps out inputs, outputs, and outcomes in a way which allows quantitative data to be entered to calculate the SROI ratio.
Implementation cost	Conceptualised as the direct cost impact of an implementation attempt. Three components of cost are generally considered: (1) costs of initiative care models; (2) costs of the implementation strategy used; and (3) varying costs of delivery by setting.
Mechanism	Refer to the underlying entities, processes or structures which operate particular contexts e.g. a specialist nurse in busy clinic engaged with PROM development, an online app designed to use 'on the go'/ 'fit into busy lives' are hypothetical examples of mechanisms. There are no limit to the number of mechanisms in a given context.
Middle range theory	A more general explanation drawn from a range of programme theories. A middle range theory could be adapted for use in many different contexts – it is not dependent on a specific disease condition.
Outcome	Refer to the results of an intervention, whether intended or not. E.g. an intended outcomes of routine PROM collection is reduced symptom burden for patients. Hypothetical unintended outcomes could include: more effective use of NHS services; a perception of surplus 'routinely collected patient data' exasperates clinical professionals; or highlights gaps in NHS data analysis and data linking capacity.
Penetration	Otherwise referred to as coverage/saturation, captures the integration of initiatives within settings (e.g. number of initiatives adopted at a site, number of patients utilising a service, number of health professional providing a service).
PROM	Patient Reported Outcome Measures. These are validated questionnaires that patients are requested to complete at various points as part of their routine care. We provide examples in the appendices.
Prudent healthcare	A Welsh Government adopted strategy defined by 4 overall principles: Public and Professionals are equal partners through co-production; care for those with the greatest health need first; do only what is needed and do no harm; reduce inappropriate variation through evidence-based approaches.
Programme theory	An explanation of what a particular programme of work is intended to deliver/improve/change. E.g. Routine PROM collection in cataracts aims to identify patients who will benefit from surgery and use these data to triage patients to more appropriate interventions which may have better patient outcomes.
Realist evaluation	A theory-driven method used to understand the contexts, mechanisms and outcomes underpinning a programme, thereby providing an explanation of 'what works, for whom, and in what contexts'. Understanding the way that a programme operates is essential for scaling-up activities.
Retroductive analysis	Retroduction refers to the identification of hidden causal forces that lie behind identified patterns or changes in those patterns. It asks the question: 'why do things appear as they do? Retroduction uses both inductive and deductive logic, as well as insights or hunches. It involves thinking through what causal powers might be at work in producing observed patterns or changes in patterns. It is underpinned by a belief that an understanding of causation cannot be achieved using only observable evidence. Retroductive theorising requires that inquirers use their common sense, intelligence, expertise, and informed imagination to build and test theories about underpinning causal processes.

For peer review only

Social Return on Investment (SROI)	A method used to measure and value outcomes that matter to the people and organisations (stakeholders) who experience them. It incorporates social, economic and environmental value, and produces an SROI ratio which tells us '£x of social value is generated for every £1 invested in the programme under evaluation'.
Social value	A concept which encompasses social, economic and environmental value.
Stakeholder	People or organisations directly affected by the activities of the programme under evaluation
Sustainment	Maintenance of fidelity measurements in an ongoing, stable manner. Emphasises the integration of the initiatives within an organisation's culture. Attaining long-term viability is considered the final stage of diffusion and dissemination within an organisation.
Theory of change	A type of logic model which describes the relationship between inputs, outputs and outcomes/ contexts, mechanisms or outcomes.
Value Based Healthcare (VBHC)	Value Based health Care is an approach which shifts the focus of healthcare onto the outcomes that matter for patients. It is realised when we achieve the best possible health outcomes for our population within available resources. It is a Welsh Government Policy and is linked to all major health and social care policy contexts. One way to support VBHC are PROMs.

Value in health care is realised when we achieve the best possible health outcomes for our population within the resources that we have available (8).

Demonstrating high value care in "real world" settings is challenging since most healthcare systems (including the NHS) have traditionally focused on recording processes (mainly access to care) rather than patient outcomes. The emergence of VBHC as a concept has led to a renewed interest in recording patient reported outcomes (PROMs) as part of routine care (9). PROMs differ from clinician recorded outcomes since they measure patients' perspectives on the impact of disease on their lives. To this end, PROMs have become a central feature of a VBHC approach, with organisations such as International Consortium for Health Outcomes Measurement (ICHOM) recognising the potential benefits for patients from utilising these tools in routine care rather than within the confines of clinical trials (10).

In 2015, the Aneurin Bevan University Health Board (ABUHB; an integrated Healthcare system covering a population of 600,000 in Wales, UK), set up a dedicated VBHC team with the aim of collecting PROMs data across multiple disease areas. The programme has grown exponentially and is now collecting PROMs in over 25 disease areas. In order to support collection at scale, ABUHB partnered with a software supplier (Dr-Doctor) to develop a novel electronic outcomes capture platform. The platform enables either remote or "in-clinic" collection of electronic PROMs.

One of the key aims of the programme in ABUHB is to use PROMs in direct care as a tool for driving improvement in both patient care and patient outcomes. There are a number of potential mechanisms whereby collecting PROMs may achieve this as described in Table 2.

Table 2 How routine collection of PROMs may lead to improved patient care

For peer review only

Care domain	Potential mechanisms where PROMS may improve care and outcomes
**Symptom burden**	At the individual patient level, the use of PROMs may empower patients, helping them start conversations with clinicians and focus on symptoms that matter to them. This may improve the detection of problems and support shared clinical decision making.
**Disease trajectory**	In chronic disease settings, temporal changes in symptoms can be plotted over time enabling discussions about disease trajectory and the impact of any treatments. Such data has not traditionally been visible to patients nor clinicians.
**Remote Monitoring**	The use of electronic PROMs lends itself to the development of remote monitoring, enabling virtual clinics and virtual triaging of patients awaiting treatment or review.
**Service improvement**	Feedback of aggregate PROMs data may lead to improved patient care by enabling services to identify which aspects of the service works well and which do not meet patient expectations.
**Benchmarking**	Reporting of case-mix adjusted aggregate PROM data may enable benchmarking with other organisations, thus driving improved care. Aggregate, case-mix adjusted data may also enable shared decision making by giving patients a realistic expectation of what outcomes they may achieve from a given treatment (based on aggregate data derived from similar patient cohorts).

Despite early reported benefits, the PROMs programme within ABUHB has not been formally evaluated. It is important to undertake an evaluation since there are a number of potential barriers which may impact on the goals described above, and thus limit the utility of PROMs. These barriers may include poor completion rates by patients, competing priorities preventing busy clinicians from viewing PROMs, as well as some clinicians lacking the competence or confidence to deal with symptoms uncovered by PROMs. On a service level, resource limitations may mean that appropriate timely referrals and review by other specialities (such as mental health) may not be possible and other solutions such as social prescribing may be required. Another key challenge to PROMs collection is digital exclusion. Technology has transformed healthcare (in particular post COVID); however, many people in the UK cannot access this technology. Those who are excluded are also likely to be disadvantaged socially and economically and hence likely to be the citizens who require healthcare the most (11).

Reviewing the literature for evidence in this area is challenging since most studies of PROMs have related to their use in clinical trials or national audits rather than their use as a tool in routine care. It is clear that the growing use of PROMs in clinical practice has occurred predominantly on the belief that it is intuitively ‘a good idea’ rather than an approach that is backed by an extensive evidence base (12). The relatively small evidence base that exists for their use in clinical practice is based on studies undertaken almost exclusively in mental health or oncology/palliative care services. Within these services there appears to be conflicting data as to whether PROMs support or constrain patients in sharing or raising issues with clinicians. It would appear that PROMs are useful for patients who preferred not to talk about personal

For peer review only

or sensitive issues, helping them to share information (13). It is also clear that some clinicians perceived that standardised PROMs constrained the patient–clinician relationship because they did not capture the complex and dynamic nature of patients' problems (12–18).

Given the dearth of evidence in the literature, the scale and pace at which the Programme in ABUHB is progressing, as well as the ambition of Welsh Government to replicate the work across Wales, it is imperative that the programme undertaken thus far is formally evaluated. In particular we need to fully understand (a) what is working, (b) who it is working for (c) what are the key mechanisms to maximise the use of PROMs to realise VBHC at the levels specified in recent policy contexts.

Aims and objectives

The aim is to undertake a realist evaluation and social return on investment analysis of the electronic collection of PROMS in the first adopter health board in Wales. Our objectives are to:

- Explore whether the PROMs currently collected encapsulate outcomes that matter to patients.
- Evaluate whether PROM collection improves patient care in Parkinson's disease, epilepsy, heart failure and cataract services. Improved patient care might be
  i) more timely,
  ii) closer to home,
  iii) direct referral to relevant health professionals,
  iv) avoid unnecessary hospital visits,
  vi) prevent unplanned admission.
- Identify potential small-scale changes as part of continuous improvement, including service re-design, and improved use of healthcare utilization.
- Measure the social value of integrating PROMs in routine data collection.
- Develop logic models identifying the inputs required for clinicians to utilise PROMs in decision-making, the context, mechanisms of change, and the potential intended/unintended impacts.
- Better understand and develop ways to overcome any barriers associated with electronic PROMs collection, in order to avoid excluding cohorts of people i.e. explore whether the shift to digital collection of PROMs excludes some communities, thus widening healthcare inequalities.

Research questions

1. What works about PROMs collection, for whom, in what contexts, and why in a VBHC context?
2. What is the social return on investment of integrating PROM collection in routine care in a VBHC context?

METHODS

A mixed-methods study comprising of a realist evaluation and social return on investment analysis. The realist and SROI analyses are complementary and will be undertaken in tandem with several key points for integration built into the study design. Combining the approaches

For peer review only

will help learn more about what is needed to achieve the goals of VBHC at scale. The study design, overarching processes and integration are illustrated in Fig.1.

Setting

Health services for people with cataracts, epilepsy, heart failure and Parkinson's disease. (Supplementary file 1) These are four service areas where PROMs have been routinely collected for a number of years. By choosing four diverse tracer services we will be able to include:

- a surgical intervention (cataracts),
- a chronic disease with a large cohort of young adults (epilepsy), and
- a chronic disease affecting a predominantly elderly and sometimes frail cohort (Parkinson's disease).
- a long-term chronic condition that is most common in older people, but can affect people at any age (heart failure)

The sample across the four tracer services is suitably big enough to address the overall research aims and manageable enough to achieve the objectives within the budget and time constraints. The individual services and their adaption of PROMS to date are described in more detail in Supplementary File 2.

Data collection and analysis

Work stream 1 (WS1)*: Rapid review, documentary analysis and stakeholder engagement to develop logic models and refine programme theories for the Realist evaluation and Social Return on Investment analysis*

Rapid review: Primary searches will be conducted in CINAHL, Cochrane CENTRAL, EMBASE, MEDLINE, PsycINFO, PubMed and Web of Science, and we will include relevant reports found via key word searches on Google Scholar. We will liaise with partners in ABUHB to identify additional relevant literature. To minimise the risk of bias, the quality of papers will be assessed using a standardised checklist such as the AACODS checklist for grey literature (19) or the CASP checklists for primary studies (20). Framework synthesis methods will be used to synthesise findings.

Documentary analysis: we will undertake content analysis (21) using NVIVO of the VBHC service specification, change and implementation documents (e.g. PROMS data, administrative patient data, audits, evaluation surveys, clinical processes and outcomes) for Parkinson's disease, epilepsy, heart failure and cataract surgery services. We will identify the key elements needed to develop initial programme theories and logic, and build an understanding of implementation processes for each tracer service

For peer review only

Engagement with key stakeholders: such as VBHC steering group members, programme designers, clinical implementers and PPI representatives will also inform the development of the initial logic models and programme theories.

Through undertaking WS1, we will uncover the underlying system dynamics (relationships between agents, their attributes and rules of behaviour, network structure, feedback loops) that influence implementation and bring about outcomes. The initial logic models and programme theories will be further developed as the study progresses and serve as the theoretical framework for the study.

Workstream 2 (WS2): Realist evaluation

We will follow the methods of Pawson (22) and Rycroft-Malone (23) and focus will shift from developing initial theories in WS1 to testing theories and refining the narrative that best explains the implementation of VBHC and observed outcomes for people in the tracer services. We will explain the circumstances (context) and mechanisms that drive outcomes (CMOs) in each tracer service. CMOs will be mapped against the predetermined programme theory to determine what is and is not working and the mechanism of action to achieving the observed outcomes (positive and negative).

We will continue to engage with key stakeholder groups and PPIs.

We will conduct realist exploratory semi-structured interviews with key people in implementing PROMs such as VBHC network staff, local healthcare professionals in the tracer services, patients and carers, and combine with quantitative process data. PROMS outcome data and how they are valued will be used to test and refine CMOs constructed. We will also ask patients for their NHS number to cross check against anonymized PROMS data to identify if they have received and returned PROMS as anticipated. Interviews will be recorded, transcribed and uploaded into NVIVO for analysis.

Consistent with realist approaches, analysis will be retroductive in that it will oscillate between an inductive and deductive logic to multiple data sources as well as incorporating the researchers' own insights and PPI /stakeholder views of what causes something to work (causation) for the programme theories. We will read through and systematically analyse each interview transcript or fieldnote and code data of interest that helps explain why and how something is working or not in a specific context. Each stage of the iterative realist analysis will become progressively focused on CMO configurations. Overall, data will be analysed within data sources (documents, interviews, process and outcomes, and service evaluation data), and with key stakeholders, PPIs and steering group members over time, and then explanations will be developed across the data, with attention to the realist task of uncovering contingencies and conditions i.e. the relationships between factors that explain ABUHB's approach to implementation of PROMs, and the conditions in which they operate. We will then revisit the programme theories and refine these in light of CMO data to confirm or disconfirm our CMOs, ensuring a diversity of data sources and another level of clarification. We will map the actual process of implementation (work as done) compared to how it was planned (work as imagined). We will also translate CMOs into generalisable theoretical models (middle-range theories) for implementing large-system, VBHC programmes. The

For peer review only

process will begin by presenting and defining CMOs with key stakeholders. Further testing will be undertaken by explicitly seeking disconfirming or contradictory data and considering other Interpretations that might account for the same findings.

We will review the realist findings for the tracer services (including programme theories and CMO configurations) 'vertically' to identify common thematic elements according to context, mechanisms and outcomes. Data will also be analysed across each service 'horizontally' to uncover potential generative causal patterns between mechanisms and outcomes. This process will potentially translate the specifics of implementing VBHC in the tracer services to more analytically driven generalisable theories for scaling up the benefits from these care delivery models to achieve large system transformation across Health Boards in Wales and beyond.

Workstream 3 (WS3): Social Return on Investment (SROI) analysis

We will develop an overall programme-level theory of change to establish how inputs (e.g. costs, staffing) are converted into outputs (e.g. numbers of patients seen), and subsequently into outcomes that matter to stakeholders affected by the programme (e.g. improved mental health). The social value generated by these outcomes is then estimated in a similar way to cost-benefit analysis.

SROI analysis will be operationalised through the stages outlined in the Guide to Social Return on Investment analysis (24):

1. **Establishing scope and identifying stakeholders (carried out in WS1)**

The scope of the study is to evaluate the social value generated by the use of PROMS in the VBHC programmes for the four services. Stakeholder involvement is critical to both the design and conduct of the study; particularly in relation to the development of programme theories. To identify stakeholders, we will list all potential groups who might affect or be affected by the activities of the programmes listed above.

2. **Mapping outcomes**

The next step is to identify the potential outcomes of each programme (positive or negative, intentional or unintentional). Informed by the documentary analysis and logic models developed in WS1, an impact map will be created for each service to explain the relationship between programme inputs, outputs and outcomes for each stakeholder group, and how these outcomes can generate value.

3. **Evidencing outcomes and giving them a value**

Longitudinal data on PROMs and clinical outcomes will be extracted at an anonymized, aggregated level allowing us to evaluate the relationship between PROM collection and the outcomes experienced by patients. Data will be aggregated by age, gender, and clinical severity. A benefit of using routinely collected data is that it facilitates access to a larger cohort than would be possible with prospective data collection, thus reducing research time and costs.

It will be important to define what will be considered a material change for each of the outcomes being measured. For example, increased physical activity may be an outcome that

For peer review only

patients experience as a result of their treatment; however, a *material* change in physical activity could be defined as a patient crossing the threshold from not meeting, to now meeting, the NHS recommendation of undertaking 150+ minutes of moderate intensity activity per week. Once the magnitude of change experienced by each stakeholder group has been identified through a combination of routinely collected data; the next step of the SROI analysis involves assigning a monetary value to the outcomes experienced by stakeholders using a financial proxy.

Outcomes occurring beyond one year will be discounted by 3.5% per annum to minimise the risk of over-claiming the amount of social value generated by the programmes.

The cost of inputs required to deliver PROMs in the tracer services will be identified in consultation with VBHC leads at ABUHB.

4. **Establishing impact**
Establishing impact is necessary to reduce bias and the risk of over-claiming the benefits of the programme. We will include model parameters to take into account deadweight (the proportion of observed outcomes that would have happened to stakeholders without PROMS); displacement (the proportion of outcomes that have been displaced from one sector to another); attribution (the amount of observed outcomes that can be direct attributed to the programme), and drop-off (the length of time that outcomes last for stakeholders). The model inputs for these variables may vary between stakeholder groups, so values will be established through stakeholder interviews and routinely collected data.

5. **Calculating the SROI**
Microsoft Excel will be used to create a model for running the SROI analysis. The model variables will be identified through the logic models developed in WS1 and further evolved in WS2. The parameters for attribution, deadweight, displacement, and drop-off will be derived from analysis of routinely collected data and varied in a range of one-way sensitivity analyses. The model will be populated with data obtained through the extraction of routinely collected data. The SROI ratio is calculated by dividing the total value of outcomes by the total value of inputs across all stakeholders. The resulting ratio is the amount of social value generated for every £1 invested in the programmes. In addition to calculating the base case scenario we will perform a range of sensitivity analyses to explore how the SROI ratio would be affected if various input parameters were changed, different financial proxies were used, and varying levels of outcomes were achieved to those observed in the base case. A checklist for quality assessment in SROI analysis (25) will be used as a framework to guide the reporting of the findings.

Identification of routinely collected data for sharing in WS2 and 3
We will identify what routinely collected data of specific interest related to VBHC and PROM implementation in the four services (e.g. PROMS data, administrative patient data, audits, evaluation surveys, clinical processes and outcomes) could be used in the realist evaluation and SROI. We will then develop a process to securely share these anonymized data between ABUHB and the research team.

For peer review only

Sampling

There is no minimum sample size for realist evaluation or SROI analysis as these types of studies are primarily used to develop explanatory theory rather than to detect statistical significance. Therefore we will interview a minimum representative sample of 15 patients and carers (separately or together) from each of the four tracer services in Epilepsy, Parkinson's disease, Cataract Surgery and Heart Failure (min 45 interviews), and up to 10 staff members responsible for implementing PROMs in routine practice from each of the tracer services.

Recruitment strategy

Patients, carers and consultees

We will purposively select patients according to their age, gender, ethnicity, service and whether they completed a PROM or not. We may add additional attributes and classifications to the sampling frame as appropriate. Patients will be contacted by ABUHB staff in the first instance and carers will be recruited through the patients. Routes to recruitment include:

- ABUHB will send out an invitation and study information via their internal PROMs platform.
- Routine appointments or via email, or post, including patients who are unable to access PROMs online (at home) and have the option of filling in PROMs within the clinic (supported by staff).

Staff members can support potential participants to complete and return a consent to contact form or the potential participants can self-complete and return in pre-paid envelopes. For participants lacking mental capacity a consultee will be appointed. Once a consent to contact form is received the research team will follow up to organize an interview.

Staff

Staff involved in the VBHC programme generally or in the four services specifically will initially be approached by ABUHB staff via meetings, direct mails and face to face contact. Purposive sampling and snowballing will be used to identify respondents best placed to provide information on PROM implementation and outcomes.

Inclusion Criteria

- Stakeholders involved with the implementation of PROMs and VBHC Programmes in ABUHB and where relevant other health boards in Wales.
- All professionals in ABUHB involved in the routine collection of PROMs in the tracer services.
- Patients aged over 18 in the identified services.
- Carers over 18 linked to a patient who is receiving care in the identified tracer services.
- Identified consultees for people who lack capacity to consent.

Exclusion Criteria

For peer review only

- People under 18.
- People who do not have the capacity to consent to take part in the research and for whom a consultee is not available.

Patient and public involvement (PPI)

The study was developed alongside a wide range of PPI stakeholders involved in ABUHB, the VBHC programme, third sector, and specific individuals and groups representing the tracer services. Going forward, partner organisations will have a key role in the interpretation and application of findings ensuring that outcomes have relevance and are accessible to specific needs and circumstances of underrepresented or especially vulnerable groups. This will be important as we know that PROMs have the capacity to help those with the greatest need first, but these individuals may encounter the most barriers to complete a PROM for example, disability, health literacy or socioeconomic status. Our partners can help advise on the ways identified Context Mechanism Outcomes chains may need to be modified or adapted to account for the needs of these patients in Wales.

Figure 2 maps and helps to visualise opportunities for PPI involvement and engagement with the multiple groups already established as part of the overall VBHC Programme and how we imagine PPI to help support adoption and scale-up of the study outcomes. We will follow the UK standards for patient and public involvement throughout (26).

Ethical issues

The study has full approval from Health and Care Research Wales Research Ethics Committee #5 (22.WA/0044). Box.1 outlines the ethical issues we have considered in designing this study

Box.1 Key ethical issues relevant to this study

Capacity to consent and personal consultees

Informed consent will be sought from all participants. Where there are concerns over capacity to consent, thorough discussions will be undertaken within the research team and a final decision will be made by the lead researcher regarding including participants who lack capacity (either temporarily or permanently). Where participants are found to be lacking capacity, a personal consultee may be appointed.

Large print versions of documents such as study information sheets and consent forms will be available for participants with a visual impairment (e.g. those attending the cataracts service). We will also provide audio recordings and explanations of the study and consent processes.

Risk of distress to participants, and pathways to referral if concerns are raised

The research team acknowledge that taking part in this study will mean that participants will have to think about and reflect upon their experiences, which may raise some concerns

For peer review only

for individuals. Should participants be concerned by the subject matter or by any issues raised, staff participants will be signposted to consult with their line manager or occupational health department for support and patient participants will be signposted to the relevant health professional. The research team have detailed distress protocols to follow in these situations and are experienced in collecting sensitive data around personal experiences of healthcare support and living with long term health conditions.

Potential identification of poor clinical practice and unmet need

Researchers are experienced healthcare scientists. Standard data protection and confidentiality protocols will be followed. We will only deviate from these if we recognize a clear and immediate risk to the health and wellbeing of participants. We will partner with wider social support services and signpost to these as part of the disengagement process we may also ask participants if they would like us to send a letter to the relevant healthcare professional alerting them to the fact that they have taken part in the study.

Maintaining confidentiality of professional participants

Professionals will be provided with an information sheet and consent form explaining that their participation is anonymous. If there is a risk of identification e.g. small team, recognizable quotes etc. we will group participants under 'professionals' and work to remove any content that may lead back to any one individual. Healthcare professionals working on the study are aware that there is no intention to identify participants at an individual level and rather to present information in general terms to facilitate learning and professional practice.

Dissemination and impact

As a co-productive study involving patients, clinicians, third sector partners and academics, findings will be shared on a continual basis. Outputs will be disseminated widely through patient and clinical networks, policy and academic routes. Consistent with realist and SROI methodology, we will disseminate findings to stakeholders as they become available through presentations, meetings and events. We will host an interim event with representatives from the multiple stakeholder groups to present initial outcomes and gain feedback and direction for the remainder of the study.

We will produce a lay report to include infographics and visuals supporting wider understanding of the VBHC strategic ambition.

We speculate that the outputs of the study will include:

- A deeper understanding of the impacts of the switch to online PROMs specific to the Welsh population.
- A series of programme implementation theories explaining what is needed to realise VBHC at scale.

For peer review only

- A final report which will include examples of the contextual barriers and facilitators that promote the uptake of PROMS, thus enhancing understanding of how to implement them successfully in other health boards.
- An understanding of how social value is generated to different stakeholders, leading to a greater understanding of how best to optimise services to reduce inefficiencies, improve outcomes and maximise value.
- An upskilled, adaptive and responsive workforce with greater understanding of the impact and value of incorporating PROMs data in their decision-making; a workforce equipped to handle the ever-evolving role of healthcare professionals.

List of Tables, figures and appendices

Table 1 Glossary of terms
Table 2 Ways PROMS may improve care and outcomes

Figure 1. Outline of realist evaluation with SROI integration and explanation of processes
Figure 2. Map of PPI partners and their proposed ongoing involvement throughout the study and their capacity to influence scale up

Supplementary File 1. List of PROMS used for each tracer condition
Supplementary File 2. Individual services and their adaption of PROMS to date

References

1. Healthcare costs unsustainable in advanced economies without reform - OECD [Internet]. [cited 2022 Dec 11]. Available from: https://www.oecd.org/health/healthcarecostsunsustainableinadvancedeconomieswithoutreform.htm
2. The unsustainable trajectory of global healthcare and ways forward - Copenhagen Economics [Internet]. [cited 2022 Dec 11]. Available from: https://copenhageneconomics.com/publication/the-unsustainable-trajectory-of-global-healthcare-and-ways-forward/
3. Estimating the financial cost of chronic kidney disease to the NHS in England | Nephrology Dialysis Transplantation | Oxford Academic [Internet]. [cited 2020 Jun 12]. Available from: https://academic.oup.com/ndt/article/27/suppl_3/iii73/1822662?related-urls=yes&legid=ndt;gfs269v3
4. Care Quality Commission. 2021 [cited 2022 Dec 11]; Available from: www.gov.uk/official-documents.
5. Raymond A, Bazeer N, Barclay C, Krelle H, Idriss O, Tallack C, et al. Our ageing population: how ageing affects health and care need in England. 2021 Dec 13 [cited 2022 Dec 11]; Available from: https://www.health.org.uk/publications/our-ageing-population
6. Mclaughlin L. Assessing the efficacy of co-production to better understand the barriers to achieving sustainability in NHS chronic kidney services and create alternate pathways. BMJ Open. 2021;
7. Crisp N. What would a sustainable health and care system look like? BMJ [Internet]. 2017 Sep 1 [cited 2020 Aug 9];358. Available from: http://www.bmj.com/permissionsSubscribe:http://www.bmj.com/subscribeBMJ2017;358:j3895doi:10.1136/bmj.j3895
8. Braithwaite J, Glasziou P, Westbrook J. The three numbers you need to know about healthcare: The 60-30-10 Challenge. Vol. 18, BMC Medicine. 2020.
9. Withers K, Palmer R, Lewis S, Carolan-Rees G. First steps in PROMs and PREMs collection in Wales as part of the prudent and value-based healthcare agenda. Qual Life Res. 2020;
10. Kelley TA. International Consortium for Health Outcomes Measurement (ICHOM). Trials. 2015;16(S3).
11. Marmot M. An inverse care law for our time. BMJ [Internet]. 2018 [cited 2022 Dec 11];362. Available from: https://pubmed.ncbi.nlm.nih.gov/30065009/

For peer review only

12. Greenhalgh J, Dalkin S, Gooding K, Gibbons E, Wright J, Meads D, et al. Functionality and feedback: a realist synthesis of the collation, interpretation and utilisation of patient-reported outcome measures data to improve patient care. Heal Serv Deliv Res. 2017;5(2).
13. Hall CL, Taylor J, Moldavsky M, Marriott M, Pass S, Newell K, et al. A qualitative process evaluation of electronic session-by-session outcome measurement in child and adolescent mental health services. BMC Psychiatry. 2014;14(1).
14. Annells M, Koch T. 'The real stuff: Implications for nursing of assessing and measuring a terminally ill person's quality of life. J Clin Nurs. 2001;10(6).
15. Hagelin CL, Wengström Y, Tishelman C, Fürst VJ. Nurses' experiences of Clinical use of a quality of life instrument in palliative care. Contemp Nurse. 2007;27(1).
16. Mitchell C, Dwyer R, Hagan T, Mathers N. Impact of the QOF and the NICE guideline in the diagnosis and management of depression: A qualitative study. Br J Gen Pract. 2011;61(586).
17. Fagerlind H, Lindblad ÅK, Bergström I, Nilsson M, Nauclér G, Glimelius B, et al. Patient-physician communication during oncology consultations. Psychooncology. 2008;17(10).
18. Pettersson A, Björkelund C, Petersson EL. To score or not to score: A qualitative study on GPs views on the use of instruments for depression. Fam Pract. 2014;31(2).
19. Tyndall J. AACODS checklist for grey literature. 2010.
20. CASP. CASP checklist for systematic reviews. 2018.
21. Hsieh HF, Shannon SE. Three approaches to qualitative content analysis. Qual Health Res [Internet]. 2005 Nov 1 [cited 2022 Jul 26];15(9):1277–88. Available from: https://journals.sagepub.com/doi/10.1177/1049732305276687
22. Pawson R, Tilley N. Realistic Evaluation. London: Sage Publications; 1997.
23. Rycroft-Malone J, McCormack B, Hutchinson AM, DeCorby K, Bucknall TK, Kent B, et al. Realist synthesis: illustrating the method for implementation research. Implement Sci. 2012;7(1).
24. Nicholls J, Lawlor E, Neitzert E GT. A Guide to Social Return on Investment. London; 2012.
25. Hutchinson CL, Berndt A, Gilbert-Hunt S, George S, Ratcliffe J. Valuing the impact of health and social care programmes using social return on investment analysis: How have academics advanced the methodology? A protocol for a systematic review of peer-reviewed literature. BMJ Open. 2018;8(12).
26. UK Standards for Public Involvement [Internet]. [cited 2021 Sep 6]. Available from: https://sites.google.com/nihr.ac.uk/pi-standards/home

Authors' contributions

The study concept and design was conceived by GR, AC and JN. CL, MP, CB, CJ and LM assisted in refining the study questionnaires and study design. GR prepared the first draft of the manuscript. All authors critically revised the manuscript and approved the submitted version.

Funding statement

This work was funded by Health and Care Research Wales (HCRW) RfPPB-20-1714(P). The views expressed are those of the authors and not necessarily those of NHS Wales and HCRW

Competing interests statement

GR, AC, MP, CL and CB are employed by the Health Board whose programme is under evaluation.

Acknowledgements

For peer review only

Thank you to Angie Pullen Director of Epilepsy Services, Ana Palazon Country Director Wales, Parkinson's UK Cymru and Chair of the Wales Neurological Alliance, Owen Williams Chair of the Wales Council for the Blind. Thank you also to Roon Adams, Race Equality First; Lorraine Morgan, Action for Elders; Dawn Owen, Carers Wales and Chris Hodson Gwent Citizens Panel. Key representatives from these groups have been involved in selecting the research questions, the study design (including scope and sampling), the realist model and examples of Context, Mechanism, Outcome chains of evidence from the tracer services, reviewing recruitment documents and supporting the ethics application.

Word count: 3944

For peer review only

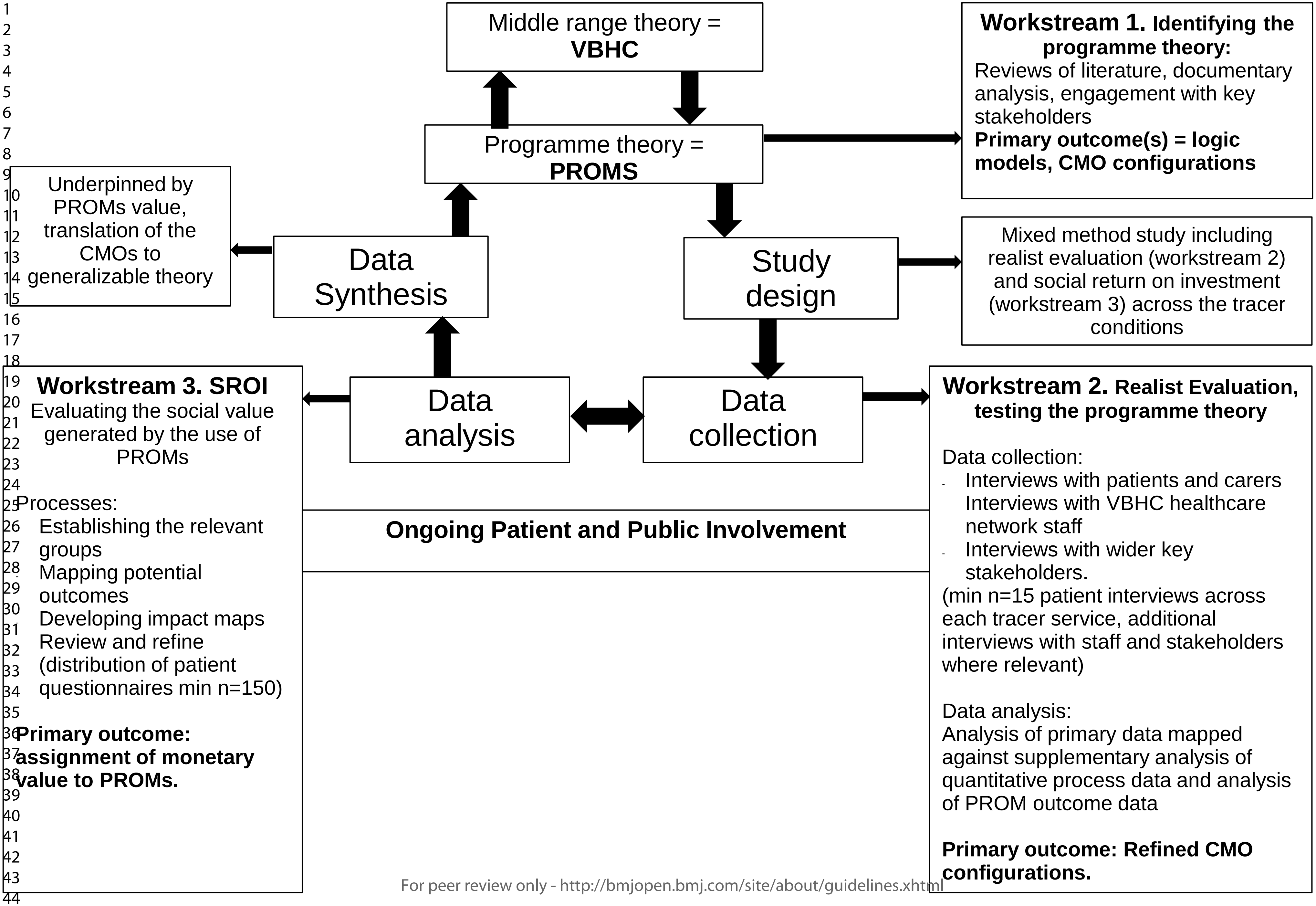

For peer review only - http://bmjopen.bmj.com/site/about/guidelines.xhtml

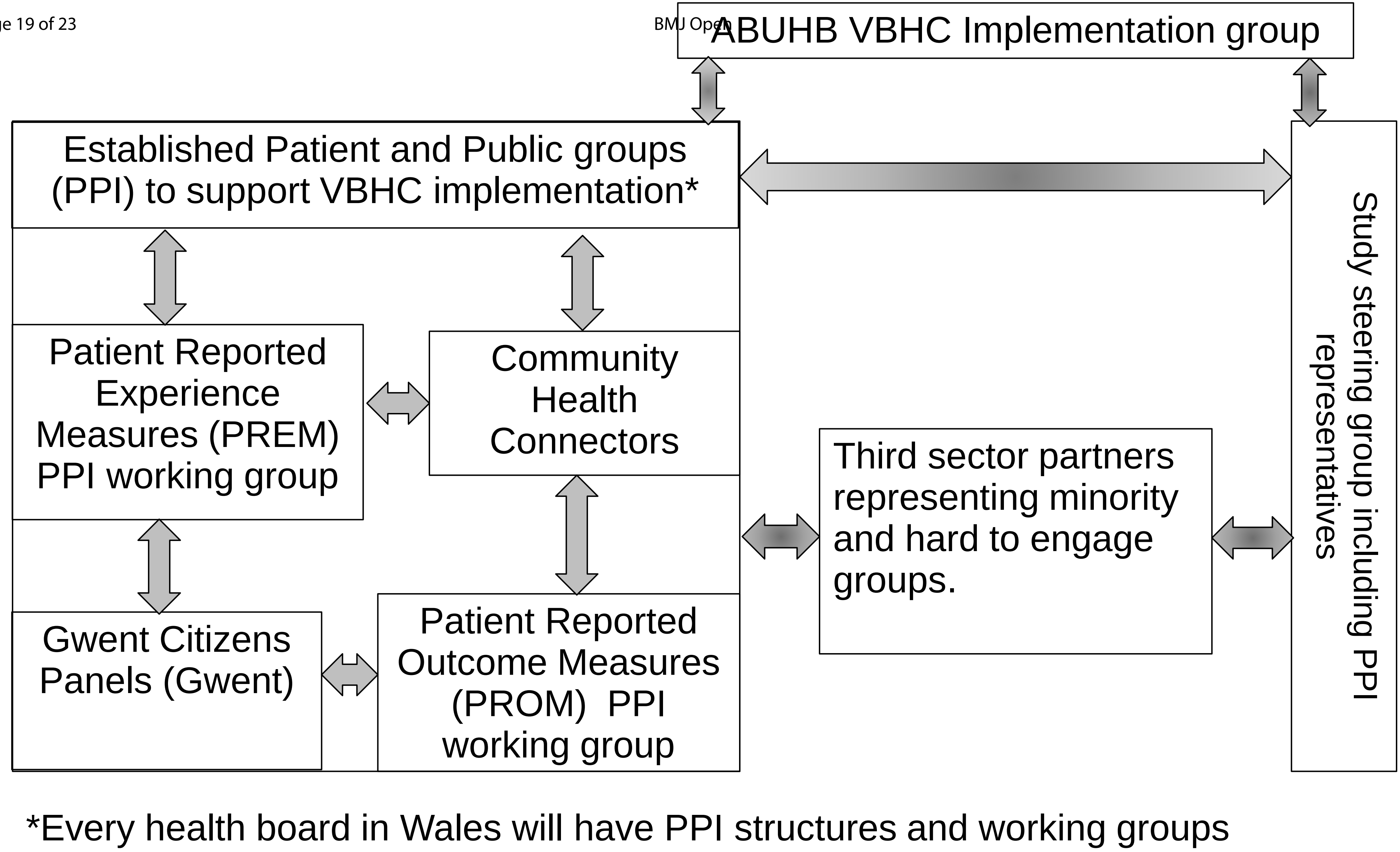
ABUHB VBHC Implementation group
Established Patient and Public groups (PPI) to support VBHC implementation*
Patient Reported Experience Measures (PREM) PPI working group
Community Health Connectors
Gwent Citizens Panels (Gwent)
Patient Reported Outcome Measures (PROM) PPI working group
Third sector partners representing minority and hard to engage groups.
Study steering group including PPI representatives
*Every health board in Wales will have PPI structures and working groups

Supplementary File 1. List of PROMS used for each tracer condition

List of PROMS used for each tracer condition

Heart failure
The Patient Health Questionnaire-2 (PHQ-2). https://pubmed.ncbi.nlm.nih.gov/14583691/

Cardiomyopathy Questionnaire (KCCQ-12).
KCCQ 12 https://www.fda.gov/media/108301/download

Epilepsy
Patients Global Impression of Change (PGIC), https://pubmed.ncbi.nlm.nih.gov/14739871/

Hospital Anxiety and Depression Scale (HADS), https://pubmed.ncbi.nlm.nih.gov/6880820/

Cataract
Cataract Questionnaire - Catquest-9SF,
https://eandv.biomedcentral.com/articles/10.1186/s40662-020-00220-4

Parkinson's Disease
Part I: Non-Motor Aspects of Experiences of Daily Living (nM-EDL),
https://pubmed.ncbi.nlm.nih.gov/23607783/

Part II: Motor Aspects of Experiences of Daily Living,
https://pubmed.ncbi.nlm.nih.gov/23791519/

The Parkinson's Disease Questionnaire (PDQ-39),
https://pubmed.ncbi.nlm.nih.gov/9351479/

For peer review only

Appendix. 2 Further explanation of the four tracer conditions and their implementation of PROMS to date

Description of the overall VBHC Programme in the adopter Health Board, and the tracer services

Initiative	Patient population	Description
Adopter health board (Aneurin Bevan UHB) implements VBHC from 2015 to present. 26 disease conditions included to date.	Health board serves approx. 639,000 patients; approx. 21% of total Welsh Population	Value Based Health Care: puts a focus on the value of outcomes achieved for patients. Value in health care is realised when we achieve the best possible health outcomes for our population within the resources that we have available.
**Tracer services**		
Epilepsy routine collection of PROMs 2017 to present	588 PROMs completed with an average completion rate of 51%	Identifying, quantifying and managing the burden of mood disorder. Identify relationship between patients mood, seizure frequency and medication to inform future treatment options
Parkinson's disease routine collection of PROMs 2017 to present.	840 PROMs completed with an average completion rate of 42%	Using PROMs to help deliver a more efficient and effective service based on disease severity and patient needs.
Heart Failure routine collection of PROMs 2018 to present	927 PROMs completed with an average completion rate of 85%	Using PROMs to reorganise the service more efficiently to reduce nurses' caseloads, reduce waiting times for patients and to focus treatment on patients' needs
Cataract routine collection of PROMs 2017 to present	3600 PROMs completed with an average completion rate of 88%	Using PROMs to redesign the referral and triage process, freeing up capacity and improving outcome by treating according to need rather than waiting times.

More detailed explanation of PROM integration to date

Tracer service 1: Cataracts

Cataract procedures are the most common operation performed in the UK, with more than 300,000 procedures carried out each year. The aim of cataract surgery is to improve quality of vision for patients and allow patients the ability to undertake everyday tasks. ABUHB receives 6000 cataracts referrals and performs more than 3,000 cataract operations every year. Demand for cataract surgery has exceeded capacity. Within ABUHB, a pilot study identified potential variation in pathways and outcomes. The VBHC team worked with the ophthalmology directorate to set up systematic collection of both clinical outcomes and PROMs. Visual acuity (ability to read letters of decreasing size) from a set distance was used as the clinical outcome. A cataract questionnaire consisting of 9 questions (CatQuest-9SF tool) was used as the validated PROM. The Catquest questionnaire explores visual impairment from a patient's perspective by asking questions about the ability to recognise faces, read newspapers or undertake hobbies. Preliminary data analysis of the first 600 cases suggested that (based on PROM scores) up to 20% of patients did not actually seem to benefit

For peer review only

Appendix. 2 Further explanation of the four tracer conditions and their implementation of PROMS to date

from surgery i.e. their visual disability did not subjectively improve after cataract surgery. Within this cohort, pre-operative PROMs scores seemed a good predictor of who would benefit the most from surgery. This has led the service to consider how pre-operative PROMs could support triage to appropriate services and inform shared decisions about surgery. We also need to better understand from the perspective of the patient and their carers what outcomes are of importance to them and whether the appropriate outcomes are being measured.

Tracer service 2: Epilepsy

The adult Epilepsy Service in ABUHB serves a population of over 6000 people with epilepsy and provides an open access service so that patients with this unpredictable condition can receive timely and responsive care. Patients can access this service via telephone or email and most patients are responded to within one working day. The service has long been aware of the rates of unmet mental health needs in the epilepsy population. There is clear data linking all forms of poor mental health with increase in seizures [23], [24]. The implementation of VBHC in ABUHB was undertaken with the aim of using PROMs to better identify and quantify the extent of anxiety and depression in people with epilepsy. Additionally, the team wanted to improve the management of mental health at individual patient level and also to track changes over time to assess whether epilepsy medication was positively or negatively affecting mood.

The Epilepsy PROM comprises questions on seizure frequency and epilepsy drugs, plus mood scores and a patient global impression change. Initial feedback from people with epilepsy revealed that the patients were overall, very happy to be asked about mood but expected feedback to them about that data and about what should happen next. The high rate of markers for suicidality uncovered by utilising PROMS scores in people with epilepsy was a deeply troubling (but potentially lifesaving) finding, but it is unclear at this stage if patient outcomes have improved.

The COVID pandemic forced the service to move to remote consultation in up to 95% of cases. COVID was also associated with an increasing demand for epilepsy services with the vast majority of people (with epilepsy) suffering with poor mood.

In terms of acting on PROMs data, in the past year the service has made:

- 18 referrals to the Mental Health team,
- 34 referrals to the Online Cognitive Behavioural Therapy (CBT) system (Silver cloud)
- 112 have been referred to online and application based mindfulness tools.
- A further 28 people have been commenced on antidepressant therapy.

PROMs are subsequently used to assess the benefits of these interventions. Please see Appendix 4 for case studies. At a local level the potential benefit to patients is clear in addition to potentially realizing the aims of VBHC, but there has been no formal evaluation to determine the mechanisms or patient outcomes and this needs addressing.

Tracer service 3: Parkinson's Disease (PD)

Parkinson's disease is a progressive nervous system disorder that affects movement. Tremors are common, but the disorder also commonly causes stiffness or slowing of movement. Within ABUHB, PROMs have been routinely collected in the PD clinic for a number of years, indeed PD was the first service to start collecting PROMs in ABUHB. The PROMs were collected in person at attendance in clinic with the PD specialist nurse, who had sight of the scores before seeing them. As well as the individual benefit of capturing all of the patient concerns, the PROMs data enables categorisation of people with PD into newly diagnosed, moderately affected and complex PD. It became clear that these patient groups had different health and social care needs with the more complex patients requiring a number of additional services

For peer review only

Appendix. 2 Further explanation of the four tracer conditions and their implementation of PROMS to date

such as physiotherapy and speech and language therapy. This led the service to begin overhauling the traditional clinic structure so that more complex patients could be stratified into a dedicated multidisciplinary clinics where their needs could all be addressed. Unfortunately, this development was paused during the COVID pandemic therapy which, as in other areas led to a wholescale switch to remote (rather than in-clinic) collection of PROMs. Of note, in spite of concerns that a more elderly cohort may not embrace remote collection, completion rates have been surprisingly high at 54%. A largescale evaluation is now needed to explore these issues and service overhaul in depth to see if patient outcomes have improved.

Tracer serviced 4: Heart Failure

Heart failure is a long-term condition that tends to get gradually worse over time. It cannot usually be cured, but the symptoms can often be controlled for many years. PROMs were introduced in the Heart Failure service in 2018, because the service was overwhelmed with patients and unable to meet demand. Patients were seen in an untimely manner and not necessarily in relation to their symptoms. The service introduced PROMs in an attempt to be more patient-focused. Previously, PROMs were applied at one hospital site using non-clinical staff to administer, but for a variety of reasons this was not sustainable. However, since the introduction of PROMS in the service, the service has developed a broader picture of care at a patient-level, of service improvement and of understanding the population needs of the service.

For peer review only
