# Supplementary material for: Protocol for a realist and social return on investment evaluation of the use of patient-reported outcomes in four value-based healthcare programmes
Source: BMJ Open. 2023 Apr 27;13(4):e072234. doi: 10.1136/bmjopen-2023-072234 (PMC10151973; doi:10.1136/bmjopen-2023-072234)
Supplement: Reviewer comments [file bmjopen-2023-072234.reviewer_comments.pdf]

PEER REVIEW HISTORY

BMJ Open publishes all reviews undertaken for accepted manuscripts. Reviewers are asked to complete a checklist review form (**http://bmjopen.bmj.com/site/about/resources/checklist.pdf**) and are provided with free text boxes to elaborate on their assessment. These free text comments are reproduced below.

ARTICLE DETAILS

**TITLE (PROVISIONAL)**	A Protocol for a Realist and Social Return on Investment Evaluation of the use of Patient Reported Outcomes in Four Value Based Healthcare Programmes
**AUTHORS**	Roberts, Gareth; Cahill, Adele; Lawthom, Charlotte; Price, Martine; Blyth, Christopher; Jones, Carys; Mc Laughlin, Leah; Noyes, Jane

This article was not externally reviewed at BMJ Open. Protocols that have been independently assessed prior to submission to BMJ Open are usually fast-tracked to publication on the grounds that further substantial changes will not be possible. This independent assessment will usually be external, independent review for both a substantial grant award from a non-commercial or government funder and ethics approval.
